# Role of PDLIM1 in hepatic stellate cell activation and liver fibrosis progression

**DOI:** 10.1038/s41598-023-38144-3

**Published:** 2023-07-06

**Authors:** Bingyu Ye, Mengli Yu, Meijuan Yue, Man Yin, Chunyan Zhang, Qiwen Wang, Xinru Ding, Wenlong Shen, Zhihu Zhao

**Affiliations:** 1 State Key Laboratory of Cell Differentiation and Regulation, College of Life Sciences, Henan Normal University, Xinxiang, 453007 China; 2 Beijing Institute of Biotechnology, No. 20, Dongdajie Street, Fengtai District, Beijing, 100071 China; 3 Institute of Genetics and Developmental Biology, Chinese Academy of Sciences, Beijing, 100101 China

**Keywords:** Cell biology, Molecular biology

## Abstract

Liver fibrosis is caused by chronic hepatic injury and may lead to cirrhosis, and even hepatocellular carcinoma. When hepatic stellate cells (HSCs) are activated by liver injury, they transdifferentiate into myofibroblasts, which secrete extracellular matrix proteins that generate the fibrous scar. Therefore, it is extremely urgent to find safe and effective drugs for HSCs activation treatment to prevent liver against fibrosis. Here, we reported that PDZ and LIM domain protein 1 (PDLIM1), a highly conserved cytoskeleton organization regulator, was significantly up-regulated in fibrotic liver tissues and TGF-β-treated HSC-T6 cells. Through transcriptome analysis, we found that knockdown of PDLIM1 resulted in a significant downregulation of genes related to inflammation and immune-related pathways in HSC-T6 cells. Moreover, PDLIM1 knockdown significantly inhibited the activation of HSC-T6 cells and the trans-differentiation of HSC-T6 cells into myofibroblasts. Mechanistically, PDLIM1 is involved in the regulation of TGF-β-mediated signaling pathways in HSCs activation. Thus, targeting PDLIM1 may provide an alternative method to suppress HSCs activation during liver injury. CCCTC-binding factor (CTCF), a master regulator of genome architecture, is upregulated during HSCs activation. PDLIM1 knockdown also indirectly reduced CTCF protein expression, however, CTCF binding to chromatin was not significantly altered by CUT&Tag analysis. We speculate that CTCF may cooperate with PDLIM1 to activate HSCs in other ways. Our results suggest that PDLIM1 can accelerate the activation of HSCs and liver fibrosis progression and could be a potential biomarker for monitoring response to anti-fibrotic therapy.

## Introduction

Liver fibrosis is a significant public health problem worldwide caused by various chronic injuries including hepatitis virus, alcohol and metabolic syndrome inducing non-alcoholic steatohepatitis (NASH)^1,2^. Further, it can progress to cirrhosis and may ultimately lead to hepatocellular carcinoma (HCC)^3^. Liver fibrosis is characterized by excess accumulation of extracellular matrix (ECM) proteins and fibrous scar formation^4^. It is now generally accepted that hepatic stellate cells (HSCs) are the major source of ECM in the pathology during hepatic fibrogenesis^1,5^. In response to injury, the two profibrotic and mitogenic cytokines transforming growth factor-β (TGF-β) and platelet-derived growth factor (PDGF) responsible for the activation of HSCs^6^. Thus, HSCs activation results in the up-regulation of the expression of α-smooth muscle actin (α-SMA) and ECM proteins, such as types I collagen (COL1A1), to produce a fibrous scar^1^. During the progression of liver fibrosis, HSCs also play an important role in the modulation of inflammation and immune response^7^. However, there is currently no effective therapeutic drug available for treatment of liver fibrosis. Therefore, it is urgent to develop novel potent therapeutic targets and biomarkers for the therapy of this disease.

PDZ and LIM domain protein 1(PDLIM1), also known as CLP36, Elfin or CLIM1, is a highly conserved cytoskeleton organization regulator and plays a crucial role in maintaining cellular homeostasis^8^. However, a number of studies over the past decade indicate that PDLIM1 is dysregulated in many tumors, such as hepatocellular carcinoma (HCC)^9^, colorectal cancer (CRC)^10^, gastric cancer (GC)^11^, ovarian cancer (OC)^12^, breast cancer^13,14^, glioblastoma^15^ and chronic myelogenous leukaemia (CML)^16^. Further, the dysregulation of PDLIM1 expression in cancers affects a series of signaling pathways, such as Hippo-YAP, Wnt/β-catenin and NF-κB signaling^8,17^. Therefore, PDLIM1 also plays an important role in cell proliferation and metastasis in the progression and development of cancers^8^. These studies suggest that PDLIM1 may be a potential therapeutic target for cancer therapy. However, the potential functions and mechanism of PDLIM1 in HSCs activation and liver fibrosis progression remains enigmatic.

Therefore, in this study, we first investigated the expression of PDLIM1 in mouse models of liver fibrosis and HSC-T6 cells. We next explored the role of PDLIM1 in regulating HSC-T6 cells activation by TGF-β treatment. Moreover, the chromatin-organizing protein CCCTC-binding factor (CTCF) was recently reported to be upregulated during HSCs activation and it was also TGF-β inducible and helped TGF-β–mediated repression of TERT transcription via interactions with β2-spectrin (β2SP) and SMAD3 in Beckwith-Wiedemann syndrome (BWS)-associated tumorigenesis^1,18,19^. However, the relationship between PDLIM1 and CTCF during HSCs activation is still unknown. We therefore also studied the expression and mechanism of CTCF after PDLIM1 interference in HSC-T6 cells. Collectively, our findings suggest that PDLIM1 may serve as a promising biomarker and therapeutic target for liver fibrosis.

## Methods and materials

### Animals

Male C57BL/6 mice (5 weeks old) were purchased from Beijing Vital River Laboratory Animal Technology Co., Ltd., Beijing, China. Mice were housed under a light: dark cycles with free access to water and food. All animal experiments were performed with the approval of the ethics committee at the Beijing Institute of Biotechnology, Beijing, China, and conformed to the relevant regulatory standards. All animal studies were completed in the experimental animal center of the Academy of Military Medical Sciences, China (license number: SCXK- (Army) 2007-004, licensed by the Ministry of Science and Technology of China).

### CCl_4_ mouse models

Mice were randomly divided into two groups (control group, liver fibrosis model group, n = 12/group). The liver fibrosis mice were generated by intraperitoneal injection of CCl_4_ in olive oil (1:3) twice weekly at equal intervals for 6 weeks (1 μL/g). Mice in the control group were injected with the same volume of olive oil. After 6 weeks, mice were sacrificed and livers were collected. Subsequently, three and four mice were randomly selected for carrying out the following experiments in the control group and the model group, respectively.

### Cell culture

The HSC-T6 cell line was acquired from Procell Life Science & Technology Co., Ltd. (Wuhan, China). HSC-T6 cells were maintained in Dulbecco’s modified Eagle’s medium (DMEM; Life technologies, Waltham, MA) supplemented with 10% (v/v) fetal bovine serum (FBS; Gibco, Grand Island, NY) with 1% penicillin/streptomycin at 37 °C in 5% CO_2_. HSC-T6 cells were activated with TGF-β (5 ng/mL, Peprotech, USA) for 0, 24, 48, 72 h.

### Masson-trichrome staining and Immunohistochemistry (IHC)

Liver tissue slices were fixed overnight in 4% paraformaldehyde and embedded in paraffin and cut into 5 μm-thick slices for histopathological evaluation. Masson trichrome staining was performed using a Trichrome Stain (Masson) Kit (Sigma-Aldrich). IHC was performed as previously described^20^. The primary antibody information is as follows: anti-PDLIM1 (Affinity, DF3003).

### Immunofluorescence (IF) staining

For cell staining, HSC-T6 cells were seeded on slides in 24-well plates and incubated at 37 °C. For PDLIM1 or α-SMA staining, cells were incubated with primary antibodies against PDLIM1 (Affinity, DF3003) or α-SMA (Affinity, AF1032) for 75 min. Finally, cells were labeled with fluorescent-labelled secondary antibodies and the nucleus was stained with DAPI. For liver tissues staining, samples were subjected to heat-mediated antigen retrieval in citric acid (pH 6.0), and blocked by 10% BSA. Slides were incubated with α-SMA (Affinity, AF1032) antibody and PDLIM1 antibody (Affinity, DF3003). The nucleus was stained with DAPI. IF was analysed using fluorescence microscopy (Olympus BX51). IF staining analysis was performed on at least three independent biological replicates.

### Western blot

Western blot was performed as previously described^20^. Antibodies used in this study were PDLIM1 (Affinity, DF3003), α-SMA (Affinity, AF1032), COL1A1 (Cell Signaling Technology, #91144), CTCF (Millipore, 07-729), TNF-α (Affinity, AF7014), IL-6 (Affinity, DF6087), p65(Affinity, AF5006), anti-p-Smad2/3 (Abcam, ab254407), anti-Smad2/3 (Abcam, ab202445), anti-p-ERK (Abcam, ab201015), anti-ERK (Abcam, ab184699), anti-p-P38 (Proteintech, 28796-1-AP), anti-P38 (Proteintech, 66234-1-Ig), anti-p-JNK (Proteintech, 80024-1-RR), anti-JNK (Proteintech, 66210-1-Ig), GAPDH (Cell Signaling Technology, #2118). Western blot analysis was performed on at least three independent biological replicates.

### RNA interference (RNAi) analysis

Detection of transfection efficiency: HSC-T6 cells at 50% to 60% confluence were transfected with small interfering RNA (siRNA) using riboFECT CP Transfection Kit (166 T) for 48 h. HSC-T6 cells activation and transfection: HSC-T6 cells at 40% to 50% confluence were activated with TGF-β at 5 ng/ml for 48 h, then transfected with small interfering RNA (siRNA) using riboFECT CP Transfection Kit (166 T) for another 48 h. The sequences are Si-PDLIM1-1 target sequence: GTCATCACAAACCAGTACA, Si-PDLIM1-2: AGGAGAAGCAAGAGTTAAA and Si-PDLIM1-3: GTGGCATCAACCTGAAACA (Guangzhou Ribobio Corporation, China).

### Total RNA extraction and quantitative real-time PCR (qRT-PCR)

The qRT-PCR was performed as previously described^20^. Total RNA was isolated with TRIzol reagent (QIAGEN) and reverse-transcribed into cDNA using GOScriptTM Reverse Transcription System (Promega). qRT-PCR was performed using GoTaq® qPCR Master Mix (Promega) on a LightCycler® 96 Instrument (Roche) according to the manufacturer’s instructions. qRT-PCR analysis was performed on at least three independent biological replicates.PDLIM1-forward: CTAGCAATGACCACCCAGCA, PDLIM- reverse: GTTGTCTACGCAGCCCTTGA; β-actin- forward: ACATCCGTAAAGACCTCTATGCCAACA, β-actin- reverse: GTGCTAGGAGCCAGGGCAGTAATCT.

### RNA-sequencing (RNA-seq)

RNA-seq libraries were prepared according to Illumina TruSeq RNA preparation protocols. Libraries quality and quantity were estimated with TapeStation (Agilent Technologies). RNA-seq profiles, generated from Illumina Novaseq 6000 (150-bp paired ends), were obtained from Novogene (Beijing, China).

### Cleavage under targets and tagmentation (CUT&Tag)

CUT&Tag assay was performed as previously described^20–22^. In brief, 500,000 cells were washed and incubated with concanavalin A coated magnetic beads (Bangs Laboratories). Cell-magnetic beads complex were resuspended in Dig-wash Buffer (20 mM HEPES pH 7.5; 150 mM NaCl; 0.5 mM Spermidine; 1 × Protease inhibitor cocktail; 0.05% Digitonin), and then the primary antibody of CTCF (Millipore, 07–729) was diluted 1:50 in Dig-Wash buffer and then incubated on a rotator overnight at 4 °C. The next day, cells were incubated with an appropriate secondary antibody (goat anti-rabbit) (1:100) in Dig-Wash buffer. Then, cells were incubated with pAG-Tn5 (Novoprotein, N259-YH01-01B) (1:200) in Dig-300 Buffer (0.05% Digitonin, 20 mM HEPES, pH 7.5, 300 mM NaCl, 0.5 mM Spermidine, 1 × Protease inhibitor cocktail). For tagmentation, bead bound cells were resuspended in 50 μl of tagmentation buffer. 1 μL of 10% SDS was then added directly into the reaction to end the tagmentation. DNA was purified by 1.5 × Ampure XP beads (Beckmann Coulter Genomics, A63882). PCR cycling conditions: 72 °C for 5 min; 98 °C for 30 s; 12 cycles of 98 °C for 10 s and 63 °C for 30 s; final extension at 72 °C for 1 min. Libraries were sequenced (150-bp paired ends) by on the Illumina NovaSeq 6000 at Novogene, Beijing, China.

### Data processing and analysis

RNA-seq: Quality control (QC) of the raw sequence data from NC and siPDLIM1 samples was performed using FastQC version 0.11.4 (http://www.bioinformatics.babraham.ac.uk/projects/fastqc/)^23^. Raw reads were first mapped to the rat rn6 genome assembly using STAR2.5.3a. The mapped reads were counted using HTSeq0.8.0 toolkit. Batch effects were removed by ComBat-seq batch effect removal algorithm (https://github.com/zhangyuqing/ComBat-seq)^24^. Differentially expressed genes (DEGs) were visualized using EnhancedVolcano version 1.1.3 and Pheatmap version 1.0.12 using three replicates of NC and three replicates of siPDLIM1. The genes up- and down-regulated with FDR < 0.05 and |log2(FC)|> 0.5 thresholds. Gene ontology (GO) enrichment analysis using the clusterProfiler package.

CUT&Tag: The reads were aligned to the reference rat genome rn6 using Bowtie2 (version 2.2.9) with the parameters: –local–very-sensitive-local–no-unal–no- mixed–no-discordant–phred33 -I 10 -X 700. PCR duplicates were removed using Picard MarkDuplicates (http://broadinstitute.github.io/picard/). Heatmaps were generated by using deepTools. Peaks were called using MACS2 (version 2.1.1.20160309) with default parameters.

### Statistical analysis

Statistical analyses were done using GraphPad Prism 6 software (GraphPad Software, San Diego, CA, USA). Data were presented as the means ± SD unless otherwise stated. Two-tailed student’s t-test was used for comparison between groups. *p* values smaller than 0.05 were considered as statistically significant: *p < 0.05, **p < 0.01.

### Ethical approval

All animal experiments were performed with the approval of the ethics committee at the Beijing Institute of Biotechnology, Beijing, China, and conformed to the relevant regulatory standards. All animal studies were completed in the experimental animal center of the Academy of Military Medical Sciences, China (license number: SCXK- (Army) 2007-004, licensed by the Ministry of Science and Technology of China). All experiments were performed in accordance with the ARRIVE guidelines.

## Results

### PDLIM1 is up-regulated in CCl_4_-induced liver fibrosis model mice

**Figure 1:**
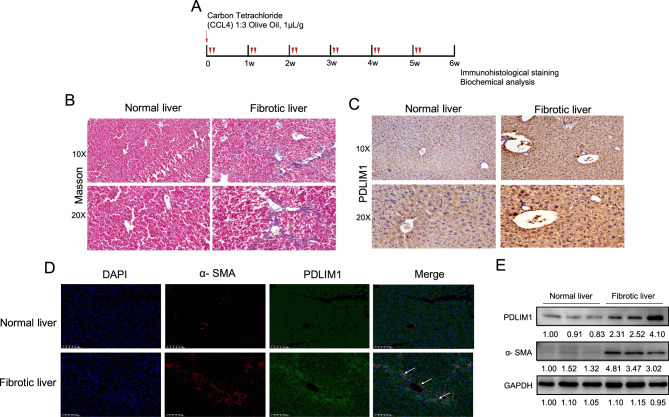
PDLIM1 is up-regulated in fibrotic liver tissues. (**A**) A schematic flow of the in vivo treatment. Mice (n = 12 mice per group) were challenged with CCl_4_ twice weekly at equal intervals for 6 weeks. (**B**) Masson trichrome staining in the livers of normal liver (n = 3) and fibrotic liver (n = 4) mice consecutively injected intraperitoneally with CCl_4_. (**C**) Immunohistochemical staining of PDLIM1 from normal liver (n = 3) and fibrotic liver (n = 4) mice. (**D**) Representative immunofluorescence images of α-SMA (red) and PDLIM1 (green) from normal liver and fibrotic liver mice. Nuclei are stained with DAPI (blue). Scale bars, 20 μm. (**E**) Western blotting analysis of PDLIM1 and α-SMA expression in liver tissues of three normal mice and four fibrotic mice. However, it should be emphasized that one of the protein extracts from fibrotic liver was degraded. Therefore, it is not represented in this figure. GAPDH was the loading control.

PDLIM1 plays an important role in cytoskeletal organization and cellular homeostasis. To investigate the role of PDLIM1 in the development of liver fibrosis, we first induced liver fibrosis in mice by CCl_4_ treatment for 6 weeks (Fig. 1A). Histopathological analysis was performed on formalin-fixed tissue after Masson-trichrome staining, tissue IF and IHC. Masson-trichrome staining revealed higher levels of collagen deposition in mouse fibrotic liver than in normal liver (Fig. 1B). Next, we showed that the expression of PDLIM1 was markedly upregulated in liver fibrosis models by IHC (Fig. 1C). Tissue IF showed that PDLIM1 co-localized with α-SMA in liver tissue of fibrosis mice (Fig. 1D). These results were also confirmed by Western blot analysis (α-SMA and PDLIM1) (Fig. 1E). Taken together, these results suggest that PDLIM1 expression is increased in liver fibrosis models.

Next, to clarify the role of PDLIM1 in liver fibrosis progression, we conducted an RNAi experiment using specific siRNA to knockdown PDLIM1 expression. To further identified the underlying mechanism, we analyzed the DEGs and signaling pathways after PDLIM1 knockdown using RNA-seq.

### RNA-seq analysis identifies PDLIM1-regulated genes and signaling pathways

**Figure 2:**
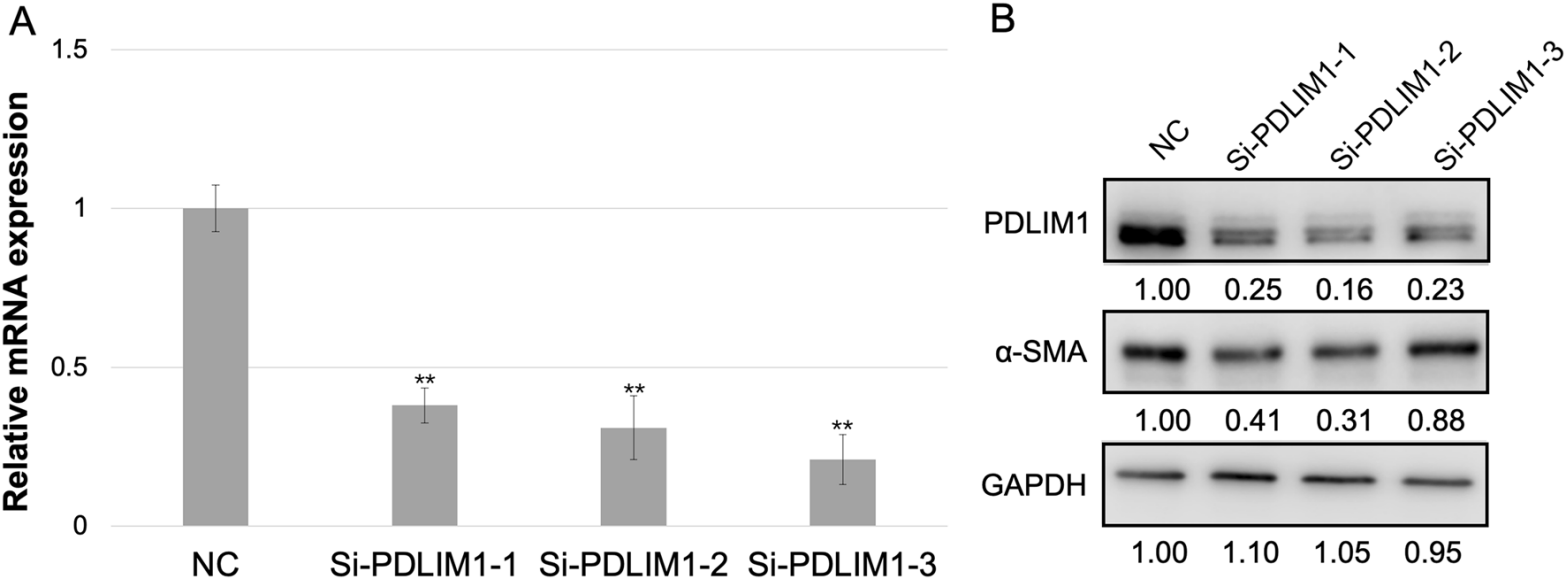
The knockdown efficiency of PDLIM1 by siRNA in HSC-T6 cells. (**A**) qRT-PCR detection of knockdown efficiency using Si-PDLIM1-1, Si-PDLIM1-2 and Si-PDLIM1-3. β-actin was the loading control. NC: negative control. (**B**) Western blotting analysis of knockdown efficiency using Si-PDLIM1-1, Si-PDLIM1-2 and Si-PDLIM1-3. GAPDH was the loading control. NC: negative control. Values are represented as mean ± SD. *p < 0.05, **p < 0.01.

Three specific siRNA (Si-PDLIM1-1, Si-PDLIM1-2 and Si-PDLIM1-3) of PDLIM1 were selected for RNAi experiment in HSC-T6 cells. The knockdown efficiency of PDLIM1 was measured by qRT-PCR and Western blot. The results indicated that the knockdown efficiency of the three fragments described above was 60–85% (Fig. 2A,B). Based on these results, we chose Si-PDLIM1-2 (hereinafter referred to as Si-PDLIM1) as the target fragment for subsequent research.

**Figure 3:**
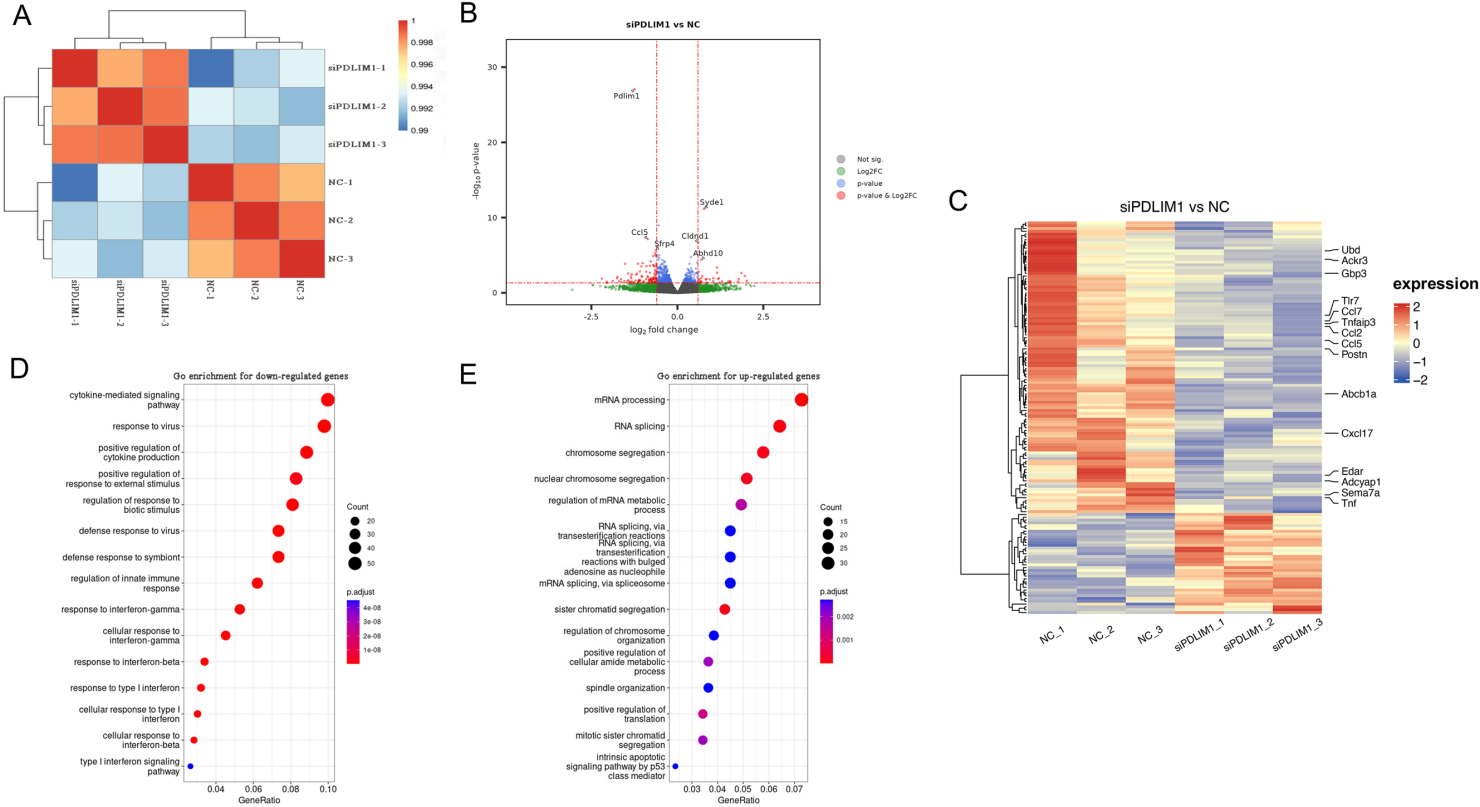
RNA-seq analysis identifies PDLIM1-regulated genes and signaling pathways. (**A**) Correlation analysis of RNA-seq data. (**B**) Differential RNA expression data were plotted using the EnhancedVolcano. (**C**) Heatmap images show DEGs of NC and si-PDLIM1 samples. NC: negative control. (**D**) and (**E**) GO analysis showing the enrichment of down-regulated (**D**) and up-regulated (**E**) genes in RNA-Seq analysis.

We then performed RNA-seq to examine the transcriptome change after PDLIM1 knockdown. Three independent biological replicates were performed for both NC and si-PDLIM1 groups. Correlation analysis indicated that replicates of each sample clustered together (Fig. 3A). After quality control, differential expression was determined based on FDR and fold change (FC) with FDR < 0.05 and |log2(FC)|> 0.5 (Table S1). The results indicated genes that were significantly down-regulated (104 genes) and up-regulated (36 genes), respectively (Fig. 3B,C and Table S2). Next, all significantly DEGs were used for gene ontology (GO) enrichment analysis. It indicated that down-regulated genes are mainly enriched in biological processes(BPs) such as cytokine-mediated signaling pathway (including Ccl7, Ccl2, Ccl5, Oas2, Cxcl9, Cxcl11, Tnf, Il1rn, Zbp1, Ackr3), chemokine-mediated signaling pathway (including Ccl7, Ccl2, Ccl5, Cxcl9, Cxcl11, Ackr3), chronic inflammatory response (including Tnfaip3, Vnn1, Ccl2, Ccl5, Tnf, Il1rn), positive regulation of response to external stimulus (including Tnfaip3, Cxcl17, Ccl2, Serpinb9, Tnf, Klrk1, Sema7a, Adcyap1) (Fig. 3D). Therefore, generally speaking, these BPs were also related to regulation of ERK1 and ERK2 cascade, I-κB kinase/NF-κB signaling, regulation of interleukin-6 production. The up-regulated genes were associated with various BPs, such as mRNA processing, RNA splicing, chromosome segregation and regulation of chromosome organization (Fig. 3E). Our transcriptional profiling results show that genes from inflammation and immune-related pathways were among the strongest coordinately down-regulated genes with knockdown of PDLIM1. These findings are consistent with the response after liver injury. We therefore hypothesize that PDLIM1 may be involved in the regulation of HSCs activation after liver injury, which remains to be further investigated.

### Effect and function of PDLIM1 on activated HSC-T6 cells

**Figure 4:**
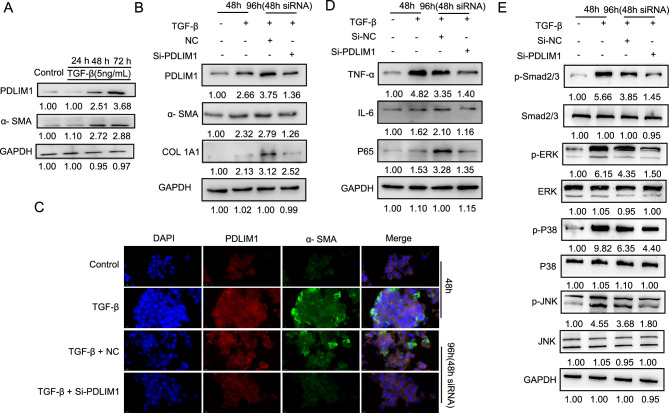
Effect and function of PDLIM1 on activated HSC-T6 cells. (**A**) Western blotting analysis of PDLIM1 expression in HSC-T6 cells treated with TGF-β. GAPDH was the loading control. (**B**) Western blotting analysis of PDLIM1, α-SMA, COL1A1 expression in HSC-T6 cells treated with TGF-β or/and si-PDLIM1. The treatment length for the siRNA was 48 h. GAPDH was the loading control. (**C**) Immunofluorescence images of PDLIM1 (red) and α-SMA (green) in HSC-T6 cells treated with TGF-β or/and si-PDLIM1. The treatment length for the siRNA was 48 h. Nuclei are stained with DAPI (blue). Scale bars, 10 μm. (**D**) Western blotting analysis of TNF-α, IL-6, P65 expression in HSC-T6 cells treated with TGF-β or/and si-PDLIM1. The treatment length for the siRNA was 48 h. GAPDH was the loading control. (**E**) Western blotting analysis of p-Smad2/3, Smad2/3, p-ERK, ERK, p-P38, P38, p-JNK and JNK expression in HSC-T6 cells treated with TGF-β or/and si-PDLIM1. The treatment length for the siRNA was 48 h. GAPDH was the loading control.

During chronic liver injury, trans-differentiation of HSCs to myofibroblast-like cells are the critical event in the development of liver fibrosis^18^. TGF-β, a potent paracrine cytokine, is widely used to induce activation of HSCs into myofibroblasts^25^. Therefore, TGF-β was added to cell culture to induce HSC-T6 cells activation, which was measured by α-SMA. The expression of α-SMA was increased with TGF-β (5 ng/mL) treatment for 0, 24, 48 and 72 h. Also, the expression of PDLIM1was elevated in a time-dependent manner (Fig. 4A). Notably, PDLIM1 knockdown in HSC-T6 cells treated with TGF-β reduced α-SMA and COL1A1 protein expression levels compared to NC group (Fig. 4B). This result indicated that the expressions of myofibroblast markers (α-SMA and COL1A1) in activated HSC-T6 cells were inhibited by knocking down PDLIM1. Next, IF staining also showed that α-SMA expression level in HSC-T6 cells treated with TGF-β was markedly increased and decreased by silencing PDLIM1(Fig. 4C). In particular, the co-localization of PDLIM1 and α-SMA were increased after TGF-β treatment, but almost disappeared by knocking down PDLIM1(Fig. 4C). This result may indicate that PDLIM1 can promote the trans-differentiation of HSCs into myofibroblasts. More importantly, to further validate the RNA-seq data, TNF-α, IL-6 and P65 were analyzed by Western blot after TGF-β treatment and PDLIM1 knockdown. It showed that PDLIM1 knockdown in HSC-T6 cells treated with TGF-β suppressed the expression of TNF-α, IL-6 and P65 compared to NC group (Fig. 4D). These results further confirmed that knocking down PDLIM1 inhibited the expression of inflammation-related cytokines. TGF-β mediates liver fibrosis and HSC activation mainly through two signaling pathways, the Smad signaling pathway and MAPK/extracellular signal regulated kinases (ERK) signaling pathway^7,26^. TGF-β induced a strong increase of Smad2/3 phosphorylation in HSC-T6 cells. However, PDLIM1 knockdown attenuated TGF-β-induced phosphorylation of Smad2/3, whereas total Smad2/3 expression level was similar between groups (Fig. 4E). In addition, PDLIM1 knockdown also attenuated TGF-β-induced phosphorylation of ERK, P38 and c-Jun-N-terminal kinases (JNK) (Fig. 4E). This result suggest that PDLIM1 is involved in the regulation of TGF-β-mediated signaling pathways in HSCs. Taken together, these results suggest that PDLIM1 plays a critical role in the HSC-T6 cells activation.

### CUT&Tag analysis identifies changes of CTCF-binding sites (BSs) after PDLIM1 knockdown

**Figure 5:**
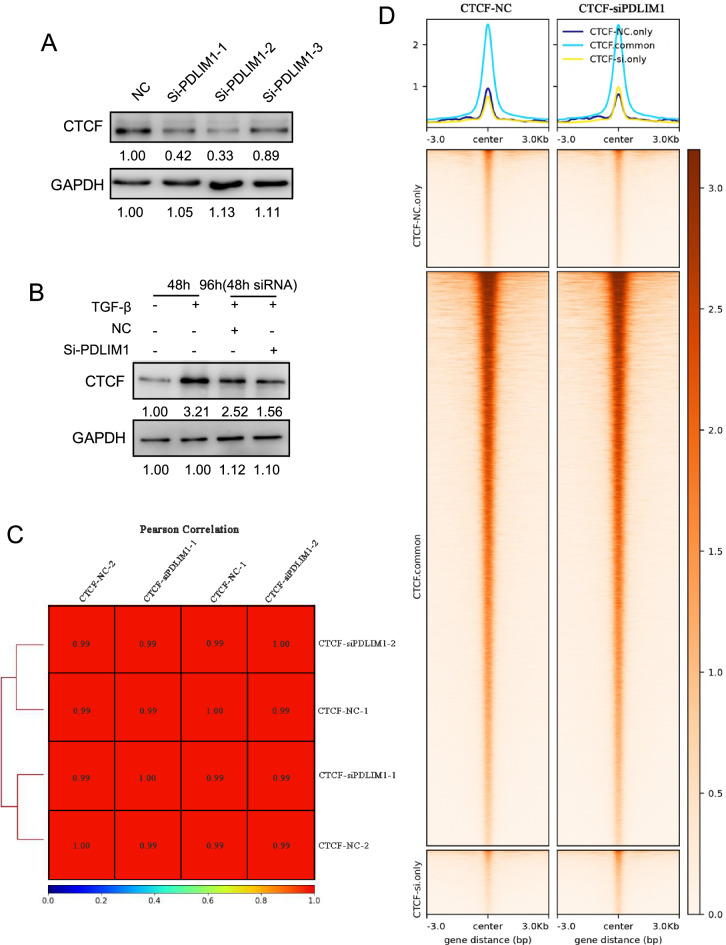
CTCF expression and BSs changes in HSC-T6 cells. (**A**) Western blotting analysis of CTCF expression in HSC-T6 cells treated with si-PDLIM1. GAPDH was the loading control. (**B**) Western blotting analysis of CTCF expression in HSC-T6 cells treated with TGF-β or/and si-PDLIM1. The treatment length for the siRNA was 48 h. GAPDH was the loading control. (**C**) Correlation analysis of CTCF CUT&Tag data. (**D**) CTCF BSs changes after si-PDLIM1.

Recent studies have shown that the expression of PDLIM1 is aberrant in cancers, such as HCC^9^, CRC^10^ and CML^16^. Moreover, CTCF is upregulated during HSCs activation^1,18^. However, whether and how CTCF plays a role in liver fibrosis and the relationship between PDLIM1 and CTCF during HSCs activation remains unclear. Western blot analysis showed that knocking down PDLIM1 partially reduced the level of CTCF (Fig. 5A). In addition, CTCF was also upregulated in HSC-T6 cells under TGF-β treatment, which was consistent with previous reports (Fig. 5B). PDLIM1 knockdown in HSC-T6 cells treated with TGF-β suppressed the expression of CTCF compared to NC group (Fig. 5B). These results implied that PDLIM1 might indirectly promote the expression of CTCF. CTCF, a major organizer of chromatin structure, plays an essential role in the establishment and maintenance of higher order chromatin structure and gene regulation^27,28^. It remains unclear whether the reduction of CTCF affects its BSs and gene regulation in HSC-T6 cells by knocking down PDLIM1. Subsequently, the CTCF CUT&Tag were carried out. Pearson correlation coefficient analysis showed biological replicates between NC and si-PDLIM1 groups were not well distinguished from each other (Fig. 5C). In other words, there was no significant difference in the peak enrichment between the NC group and si-PDLIM1 group. The partial reduction of CTCF might not significantly affect CTCF binding to its genomic sites. In order to better investigate the peak distribution difference between NC group and si-PDLIM1 group, according to peak distribution before and after PDLIM1 knockdown (FDR < 0.1, |log2(FC)|> 0.5), the peaks were divided into three groups: (1) significantly weakened (CTCF-NC only), (2) unchanged (CTCF common), and (3) significantly enhanced (CTCF-si only). The results showed that CTCF binding was not significantly affected by PDLIM1 knockdown (Fig. 5D). As an important executor of chromatin structure, the binding of CTCF to chromatin is strictly regulated. We speculate that partial loss of CTCF may affect other BPs, such as inflammation and immunity after chronic liver injury. Therefore, the role of CTCF in the process of liver fibrosis and how CTCF helps PDLIM1 promote liver fibrosis is worth exploring.

## Discussion

HSCs activation is a key event in the process of liver fibrosis. Subsequently, the activated α-SMA-positive HSCs produce ECM proteins such as type I collagen^1,29^. Furthermore, single-cell(sc) RNA-seq identifies central vein-associated HSCs are the dominant pathogenic collagen-producing cells during centrilobular injury-induced fibrosis^30^. Thus, the prevention of HSCs activation is a promising treatment strategy for liver fibrosis. TGF-β, a potent and ubiquitous profibrogenic cytokine, plays an important role in HSCs activation^7^. However, due to TGF-β has a wide range of functions, blocking TGF-β directly may have adverse effects on tissues^7,31^. Therefore, it is very important to find effective drugs against HSCs activation. In this study, PDLIM1 was upregulated in CCl_4_-induced liver fibrosis mouse models and TGF-β-treated HSC-T6 cells. Further, PDLIM1 was closely related to inflammation and immune-related pathways in HSC-T6 cells by transcriptome profiling and Western blot analysis. More importantly, PDLIM1 knockdown significantly inhibited the activation of TGF-β treated HSC-T6 cells and the promotive effect of PDLIM1 on HSC activation is mainly dependent on TGF-β signaling. Thus, targeting PDLIM1 may provide an alternative method to suppress HSCs activation.

Previous study showed that PDLIM1 acts differently in different cancers. For example, PDLIM1 promotes metastasis in glioblastoma and breast cancer, however, PDLIM1 inhibits metastasis in CRC^10,13,15^. In particular, upregulation of PDLIM1 inhibits HCC metastasis through activating Hippo signaling and loss of PDLIM1 confers poor prognosis, suggesting that PDLIM1 could be a potential biomarker for metastatic HCC^9^. In our current research, we found that PDLIM1 might be a pro-fibrotic factor in the initiation of liver fibrosis, and the dynamic expression of PDLIM1 correlated with its different roles at different stages of liver disease.

It has been reported that the TGF-β adaptor, β2SP, regulates cyclin dependent kinase 4 (CDK4) to reduce the development of hepatocellular cancer^32^. Moreover, very recently, a study highlighted a novel TGF-β and H19 (known to bind CTCF) signaling axis in tumor-initiating hepatocytes that importantly regulates hepatic carcinogenesis^33,34^. Furthermore, it has also been reported that TGF-β/β2SP/CTCF is involved in regulating tumor suppression in human stem cell disorder BWS^19^. The expression of CTCF was up-regulated during the activation of HSCs^1,18^. GO enrichment analysis of transcriptome data also showed that PDLIM1 was significantly related to the regulation of chromatin organization (Fig. 3E). CTCF is a key chromatin organizer. Therefore, we wonder whether PDLIM1 can also regulate the expression of CTCF in HSCs activation. Western blotting discovered that the expression of CTCF was down-regulated after knocking down PDLIM1 in HSC-T6 cells with or without TGF‐β treatment. We therefore suggested that PDLIM1 may indirectly regulate CTCF expression. Next, CTCF CUT&Tag analysis showed that the binding of CTCF to chromatin did not change significantly before and after knocking down PDLIM1.We hypothesized that partial loss of CTCF might not cause changes in chromatin structure. However, this part of CTCF might cooperate with PDLIM1 to participate in numerous BPs, such as inflammation and immunity after chronic liver injury. Further researches are still remains to be explored.

In conclusion, to the best of our knowledge, this is the first study to provide evidence about the important role of PDLIM1 in HSCs. Our results suggest that PDLIM1 can accelerate the activation of HSCs and liver fibrosis and could be a potential biomarker for liver fibrosis. However, the regulatory mechanism of PDLIM1 in liver fibrosis still needs more in-depth study.

## Supplementary Information

Supplementary Information.

Supplementary Table S1.

Supplementary Table S2.

## Data Availability

All sequencing data (RNA-seq and CUT&Tag) generated in this study have been submitted to NCBI with a BioProject accession number of PRJNA910644 (http://www.ncbi.nlm.nih.gov/bioproject/910644).

## Abbreviations

PDLIM1: PDZ and LIM domain protein
HSCs: Hepatic stellate cells
CTCF: CCCTC-binding factor
RNA-seq: RNA-sequencing
CUT&Tag: Cleavage under targets and tagmentation
DEGs: Differentially expressed genes
BSs: Binding sites
GO: Gene ontology
