# Supplementary material for: Role of PDLIM1 in hepatic stellate cell activation and liver fibrosis progression
Source: Sci Rep. 2023 Jul 6;13:10946. doi: 10.1038/s41598-023-38144-3 (PMC10326060; doi:10.1038/s41598-023-38144-3)
Supplement: Supplementary file 1 — Supplementary Information. [file 41598_2023_38144_MOESM1_ESM.docx]

Figure 1

PDLIM1


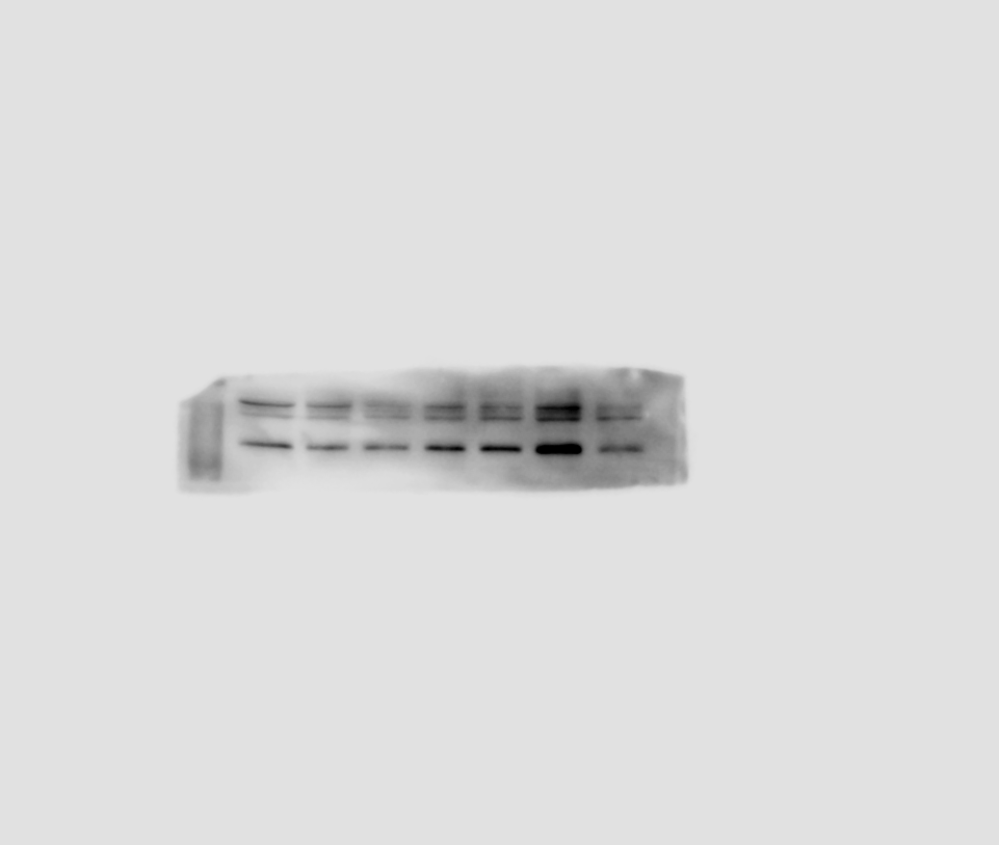


α-SMA


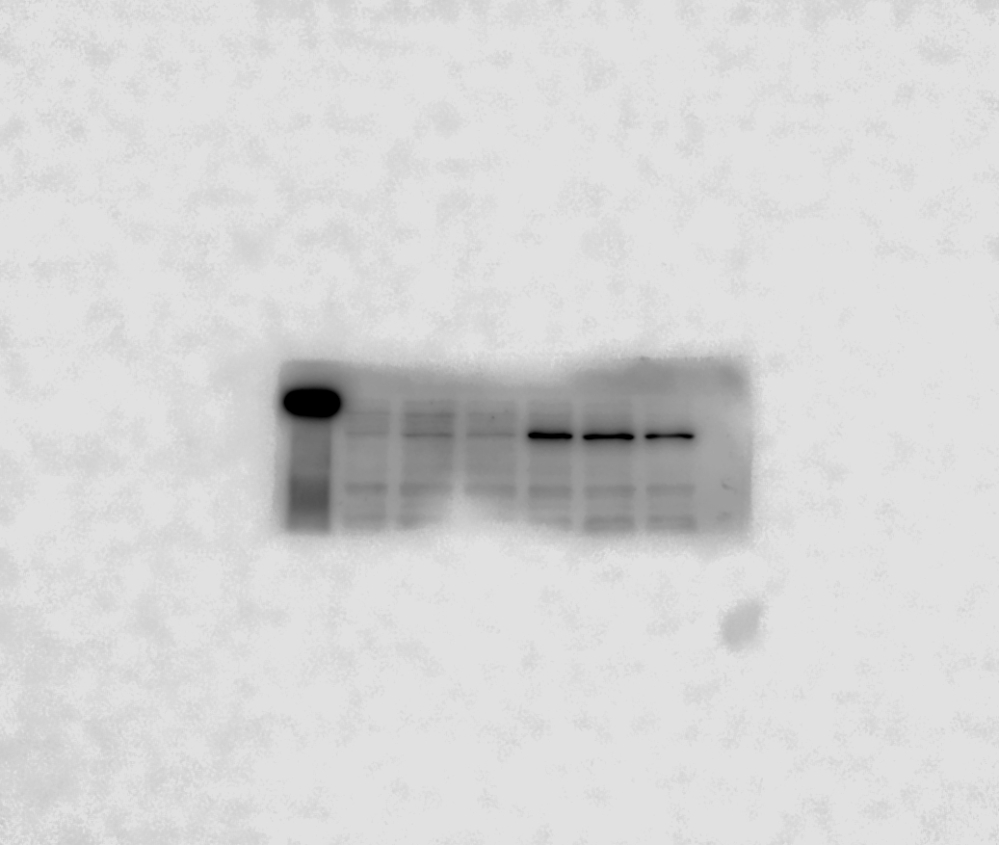


GAPDH


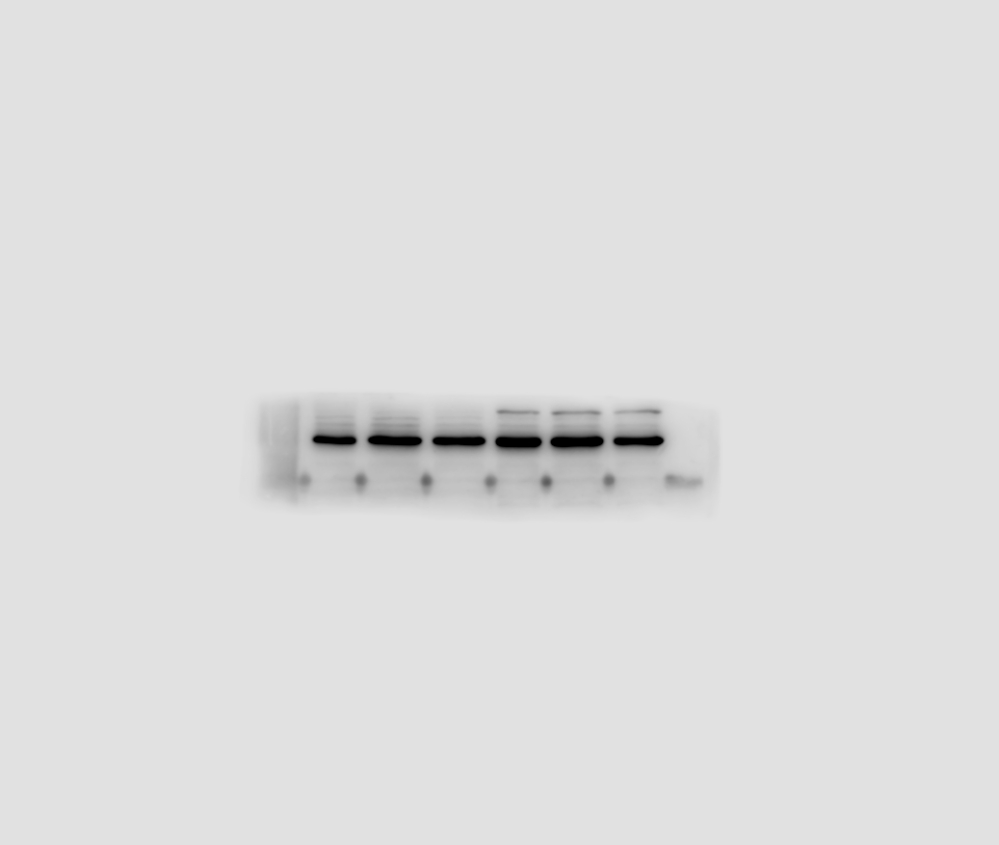


Figure 2B

PDLIM1


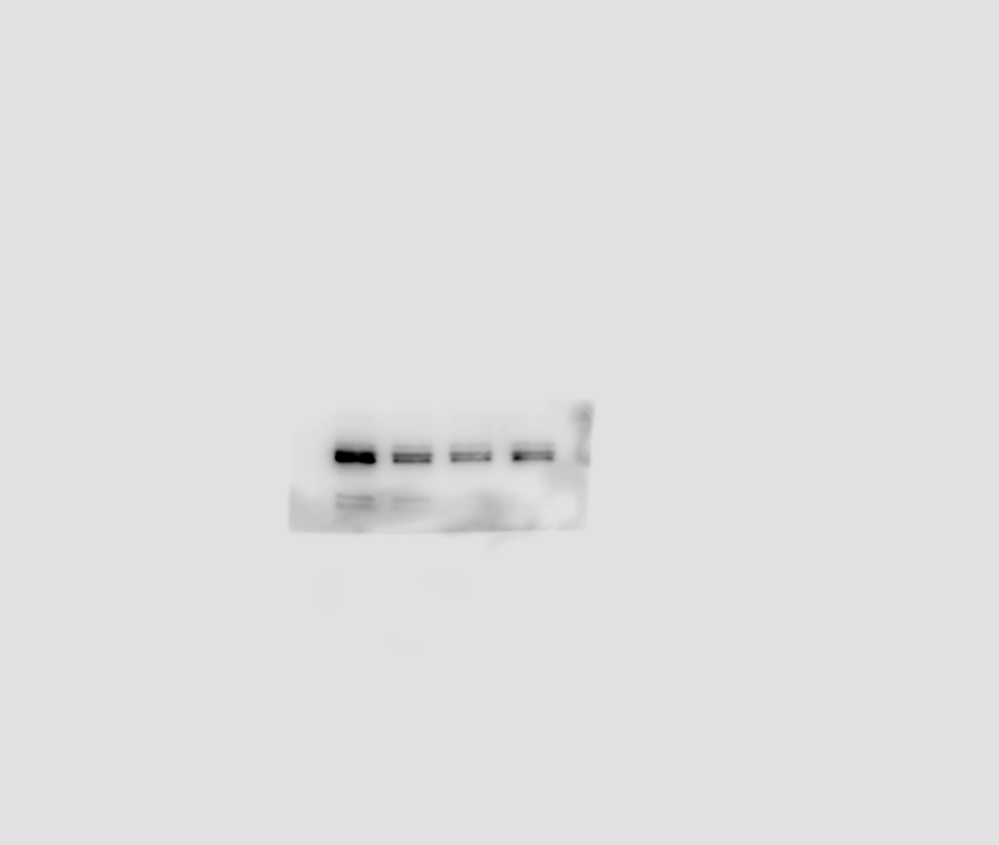


α-SMA


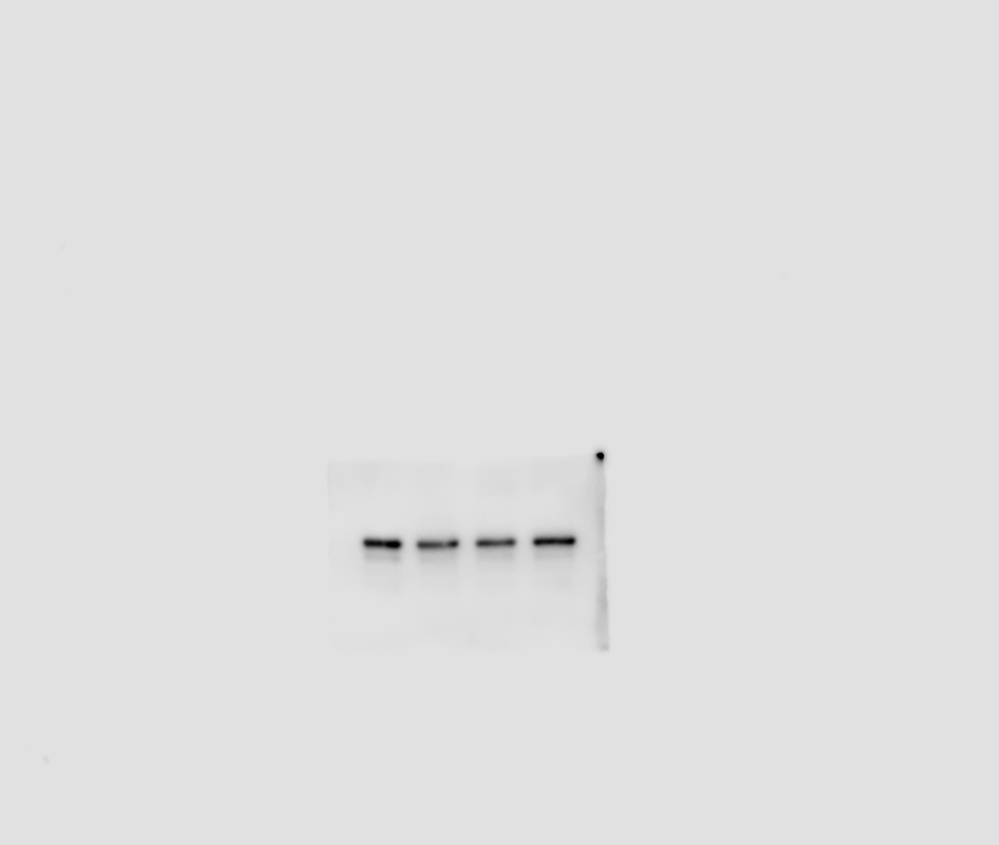


GAPDH


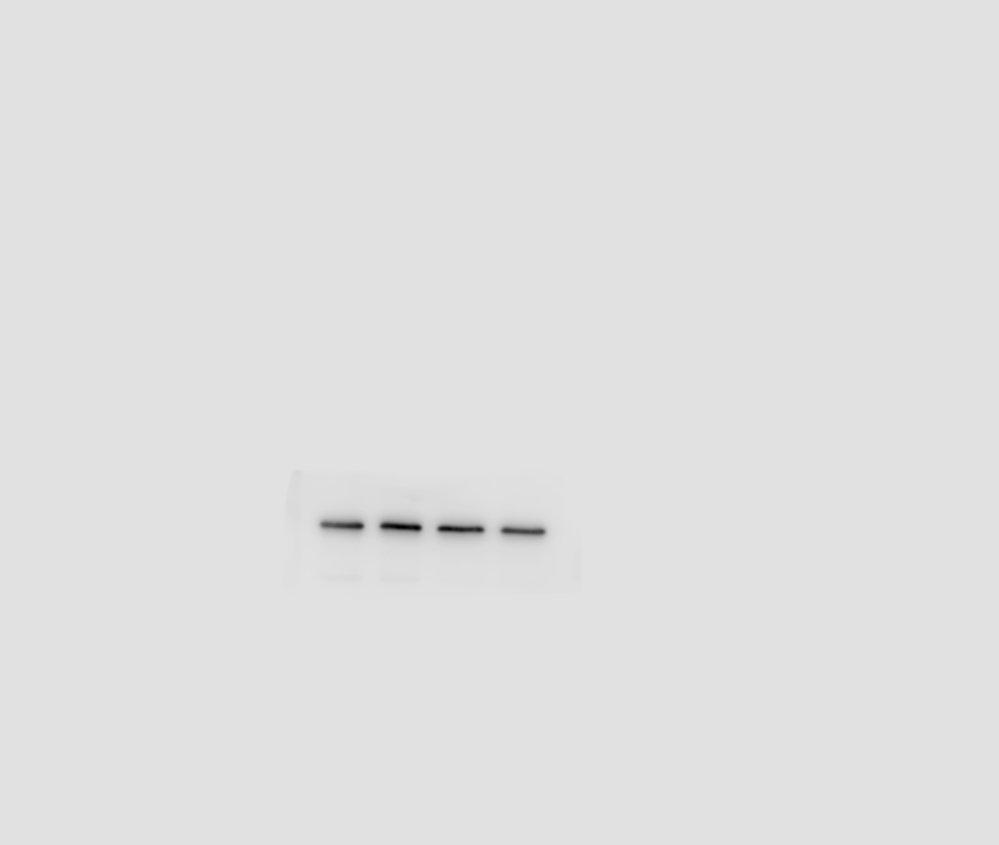


Figure 4A

PDLIM1


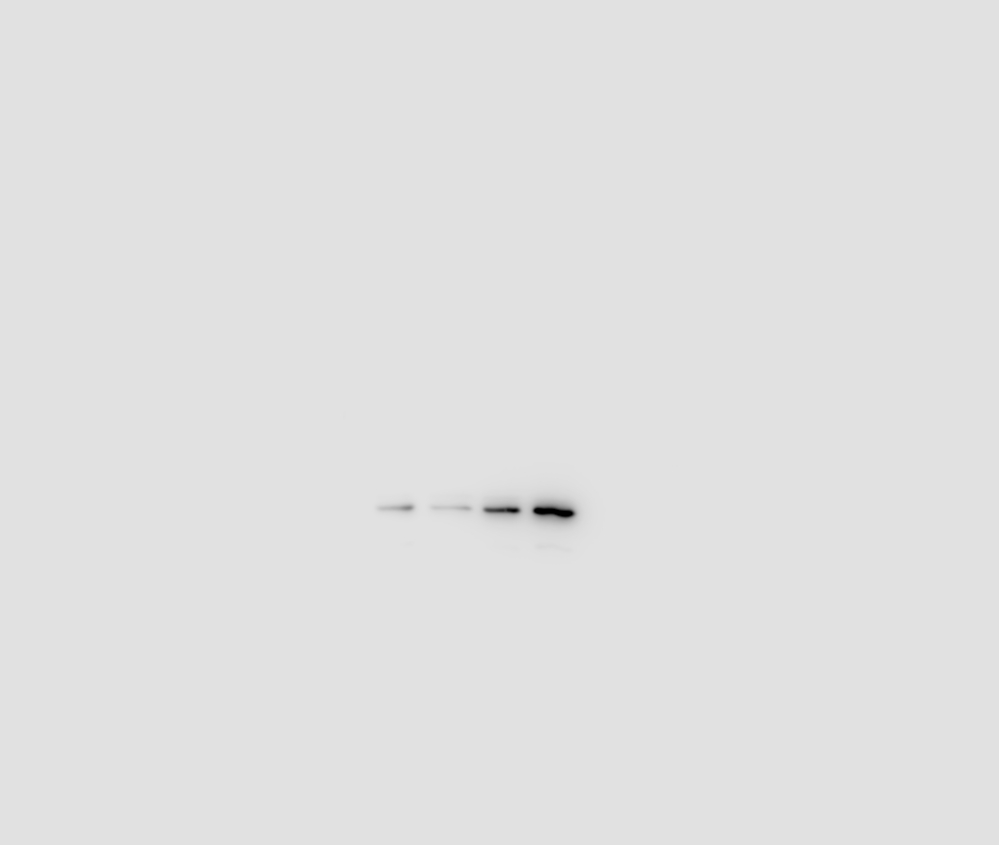


α-SMA


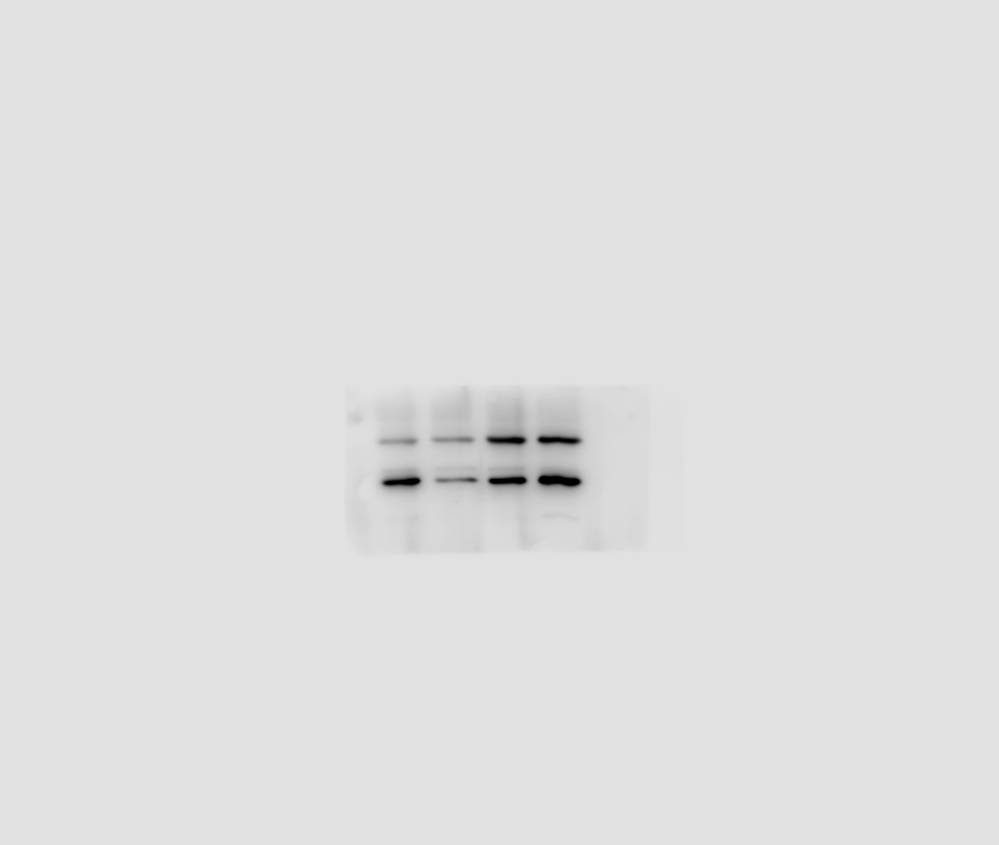


GAPDH


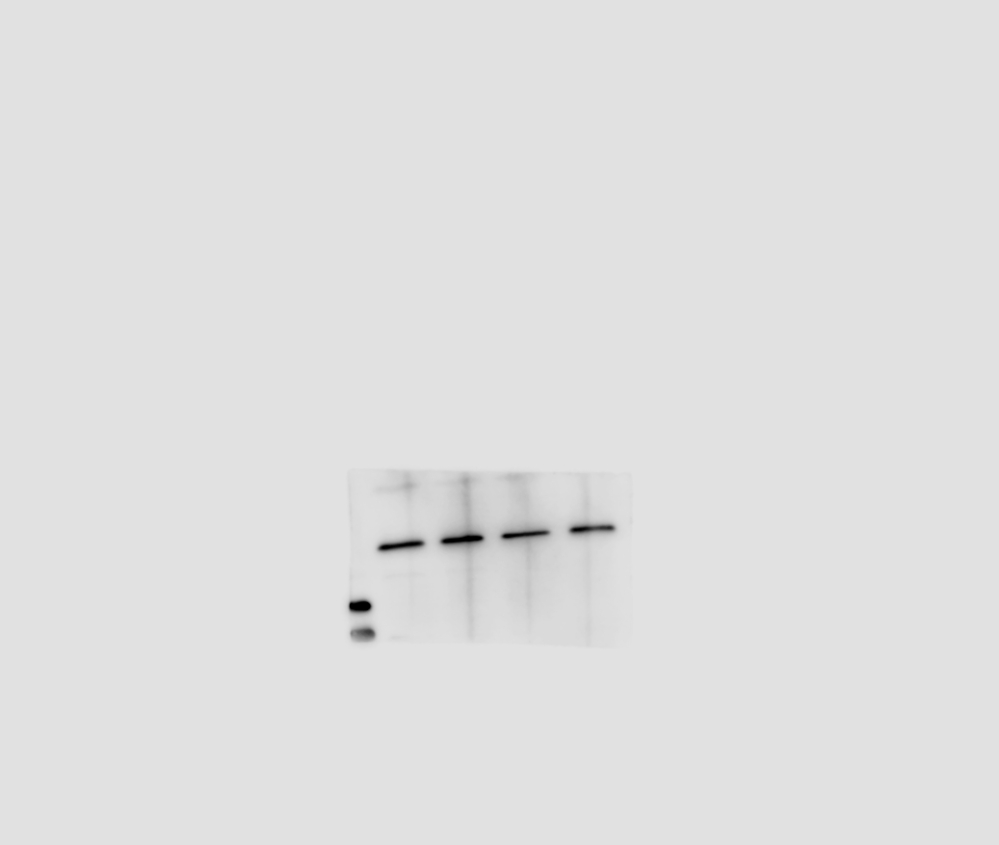


Figure 4B

PDLIM1


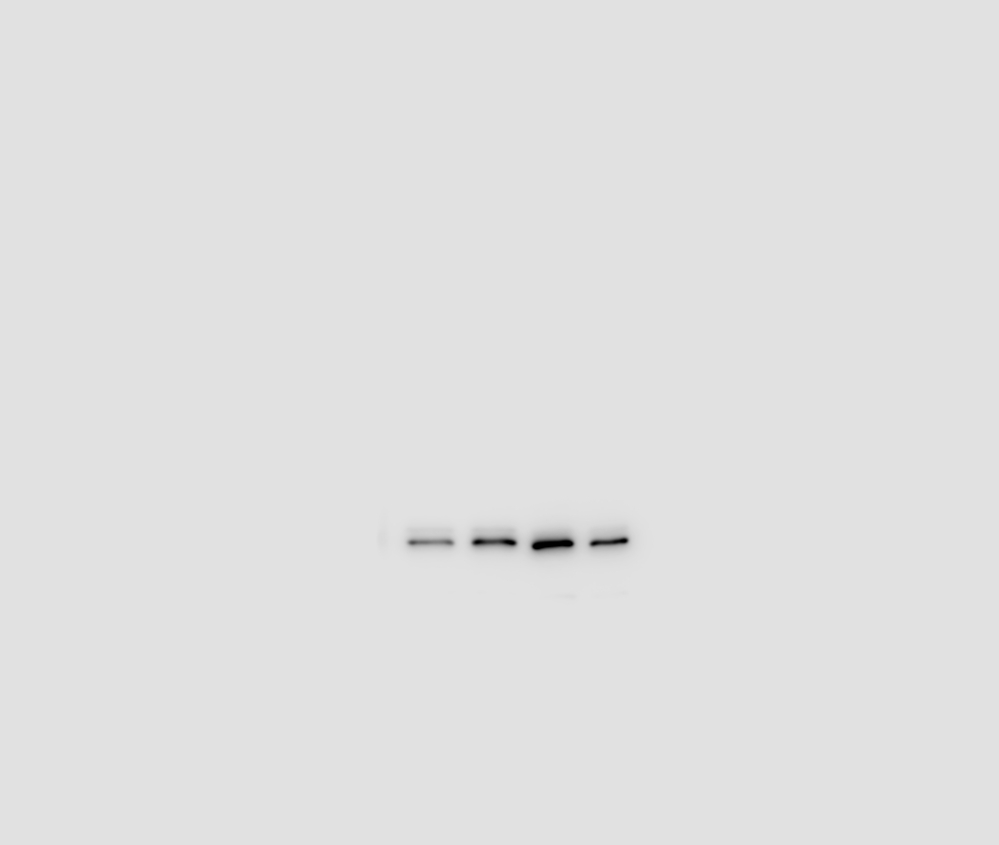


α-SMA


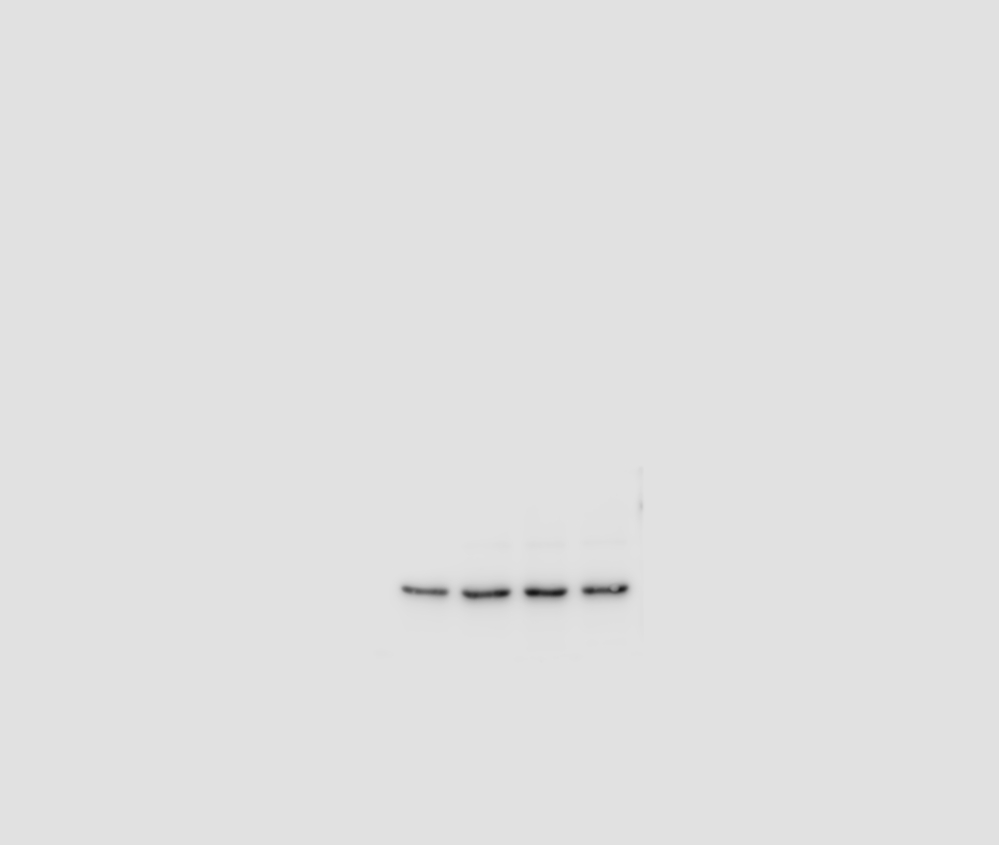


COL 1A1


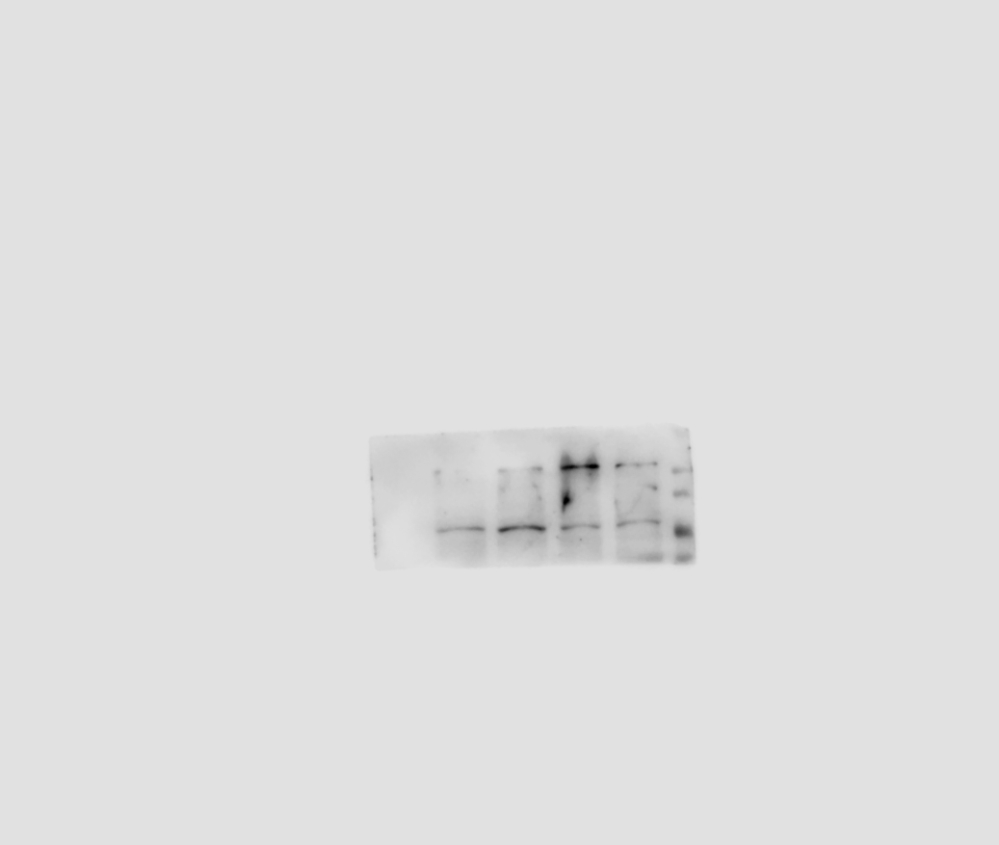


GAPDH


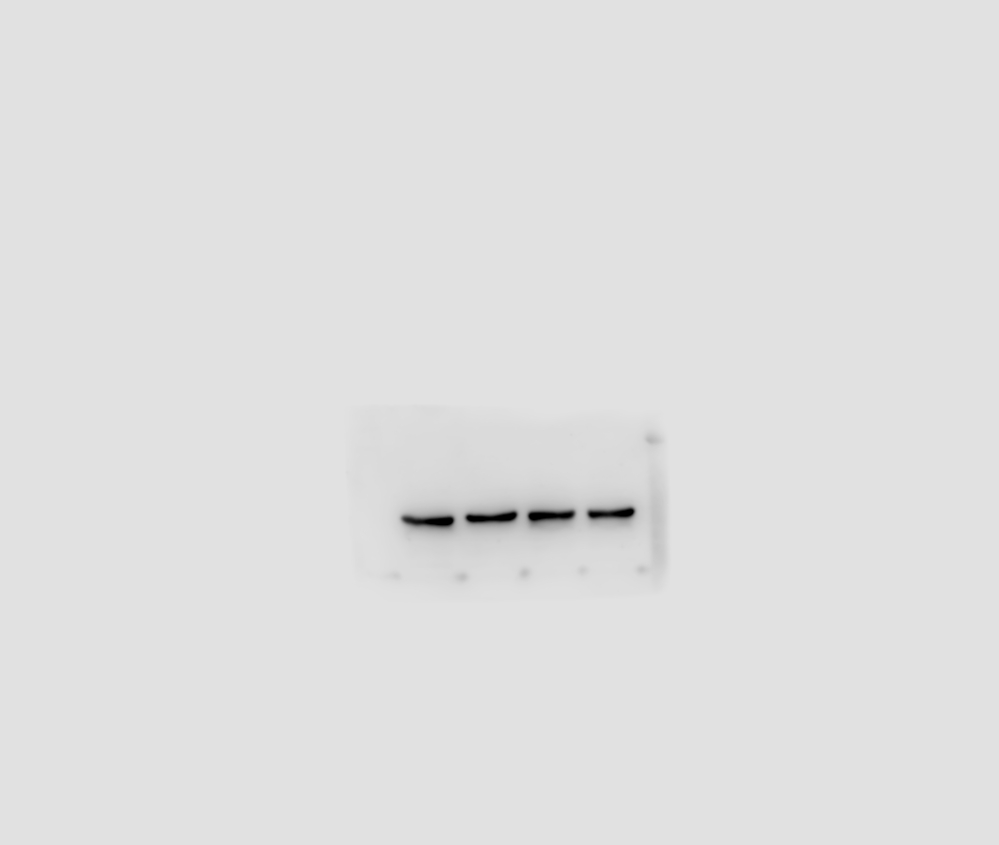


Figure 4D

TNF-α


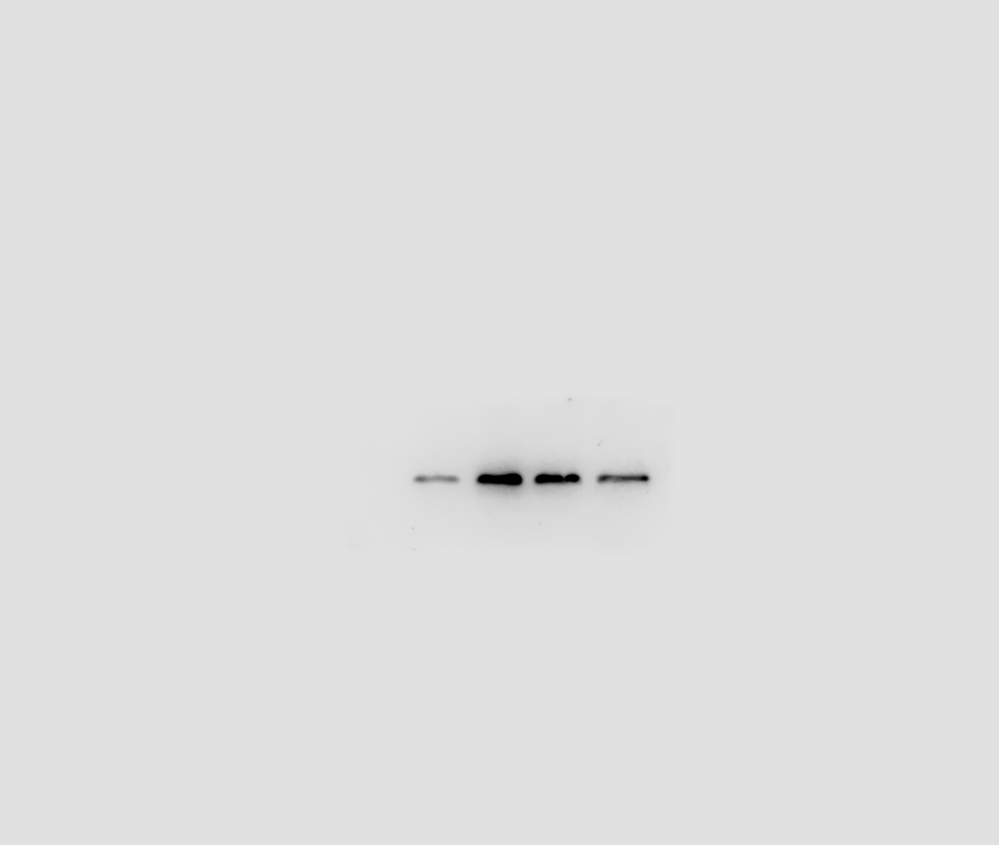


IL-6


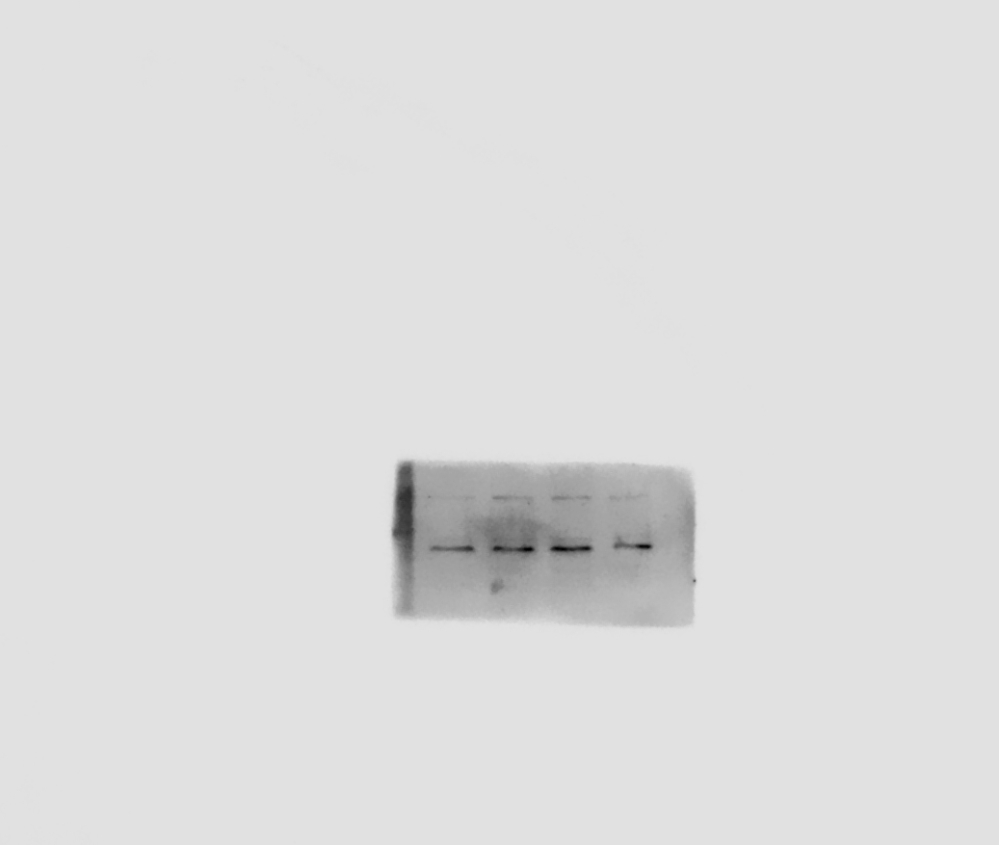


P65


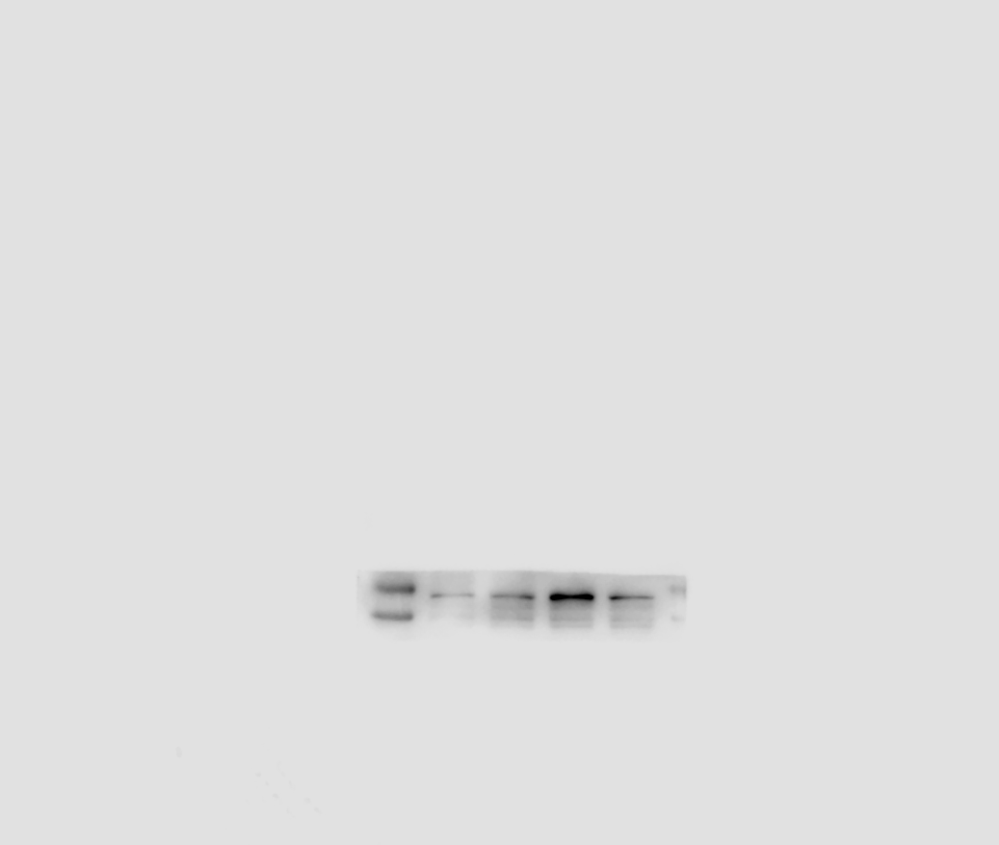


GAPDH


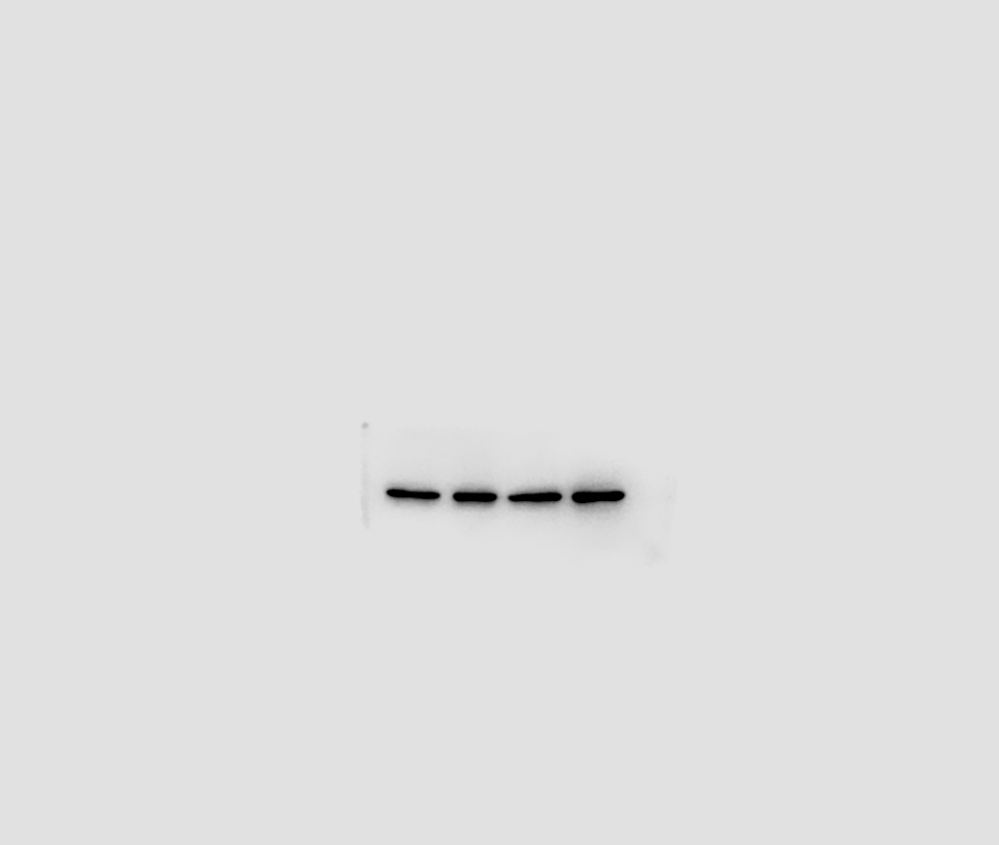


Figure 4E

p-Smad2/3


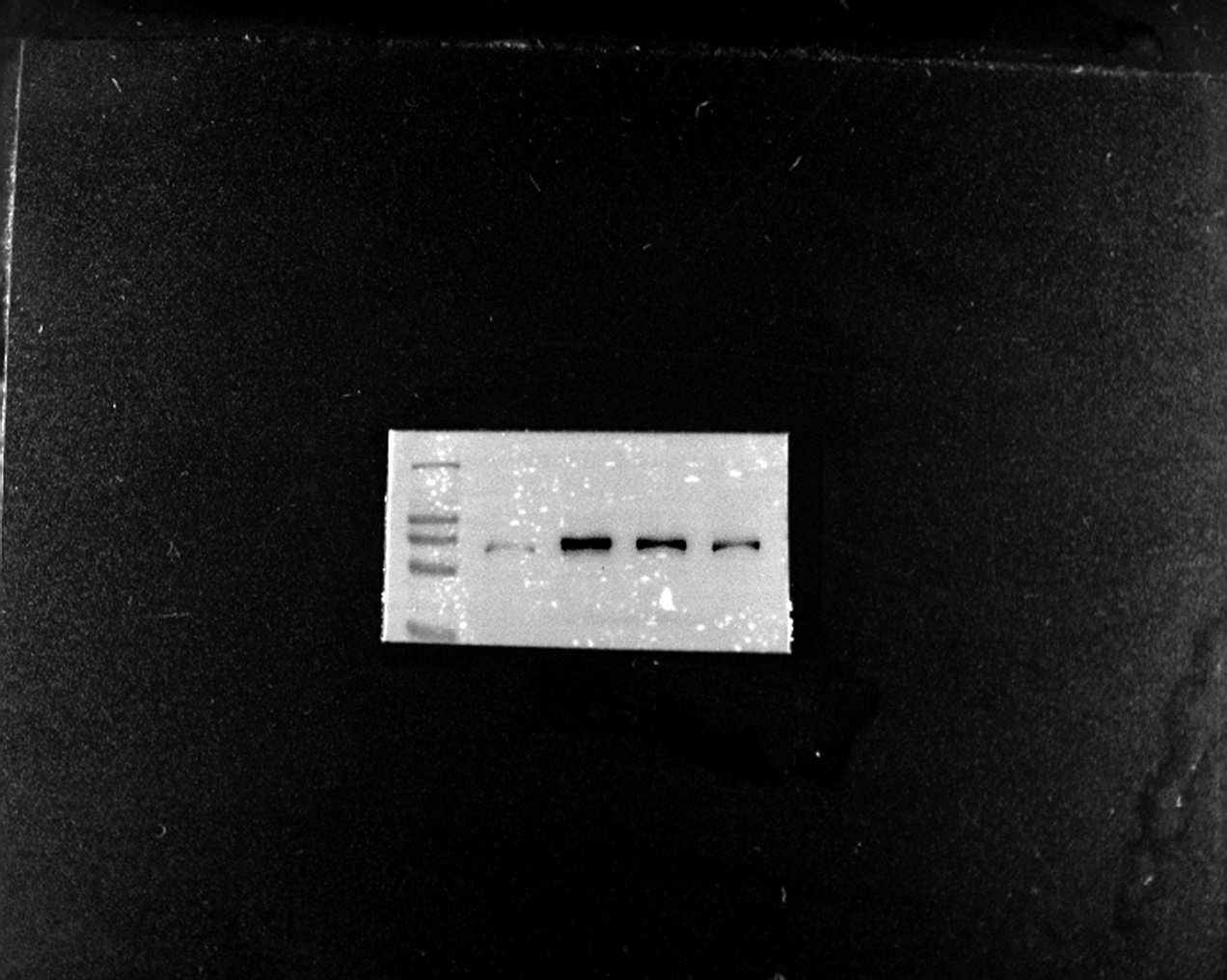


Smad2/3


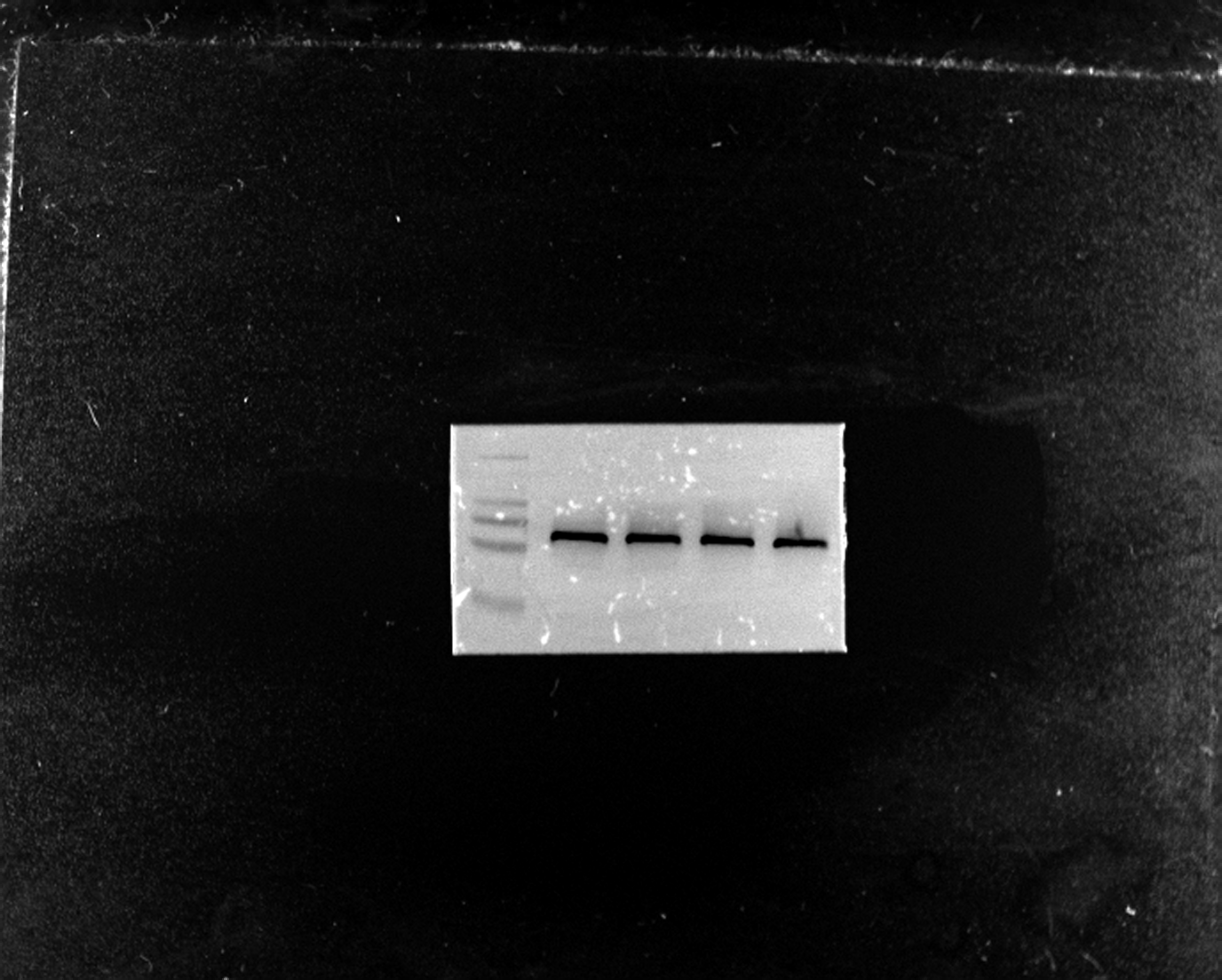


p-ERK


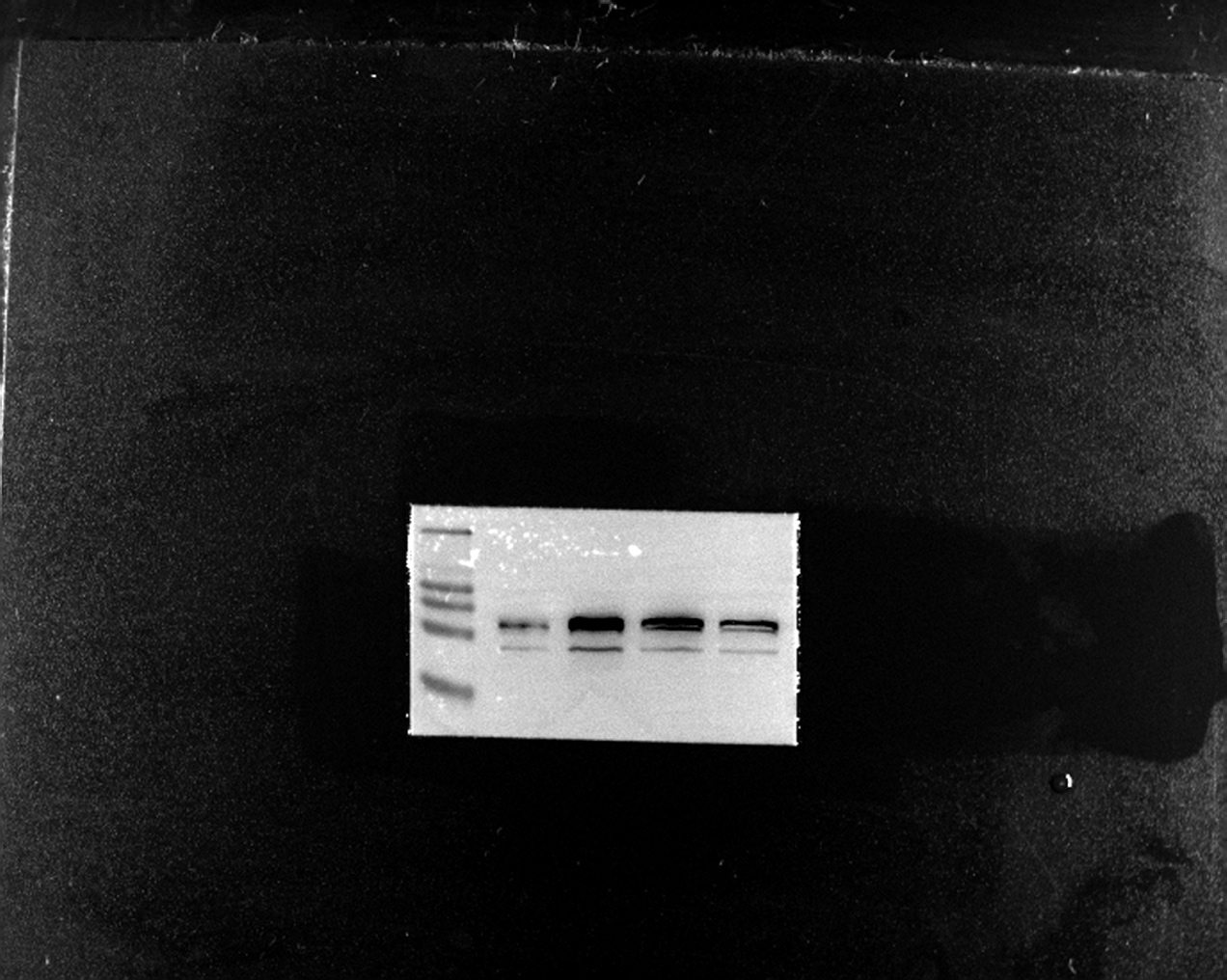


ERK


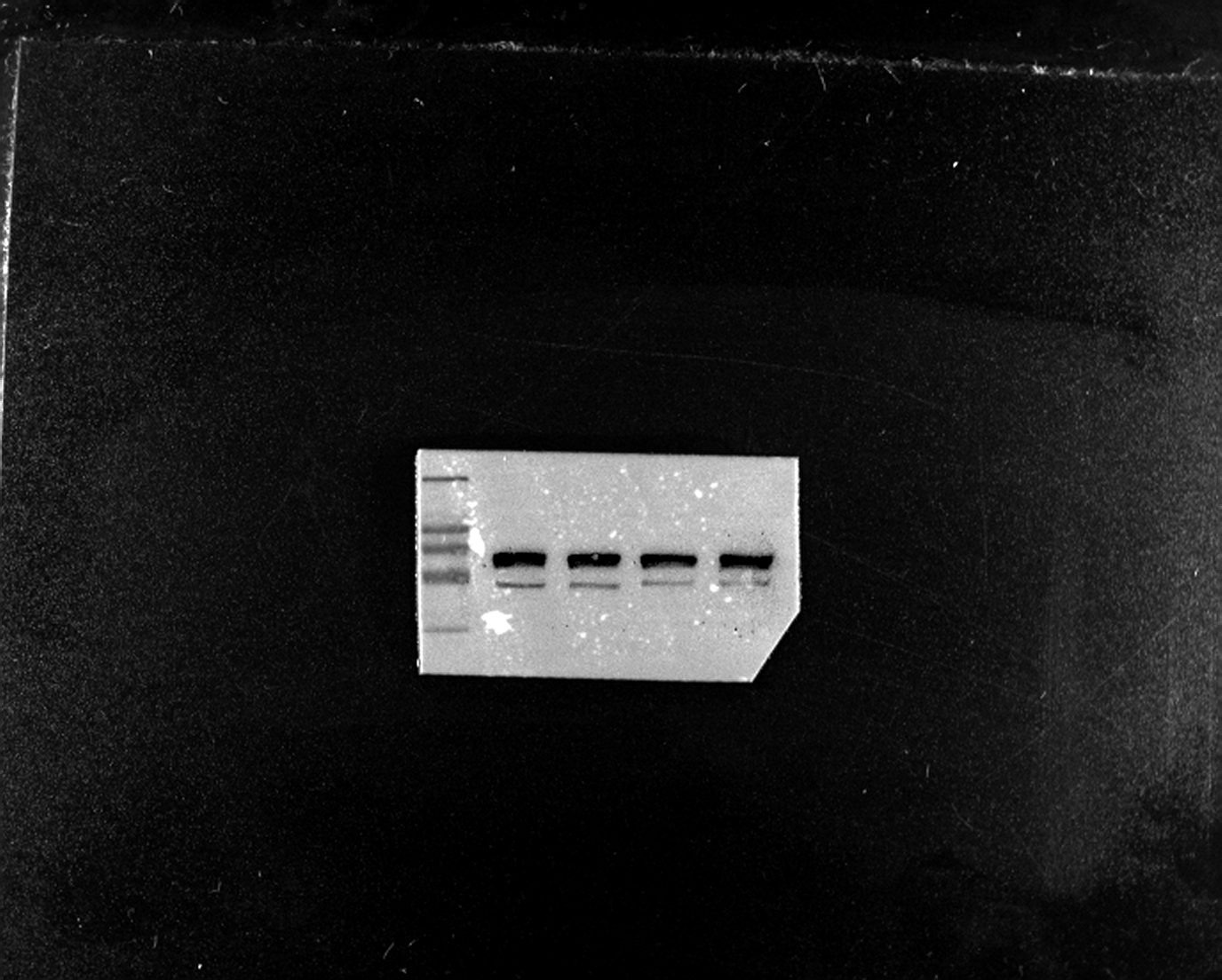


p-P38


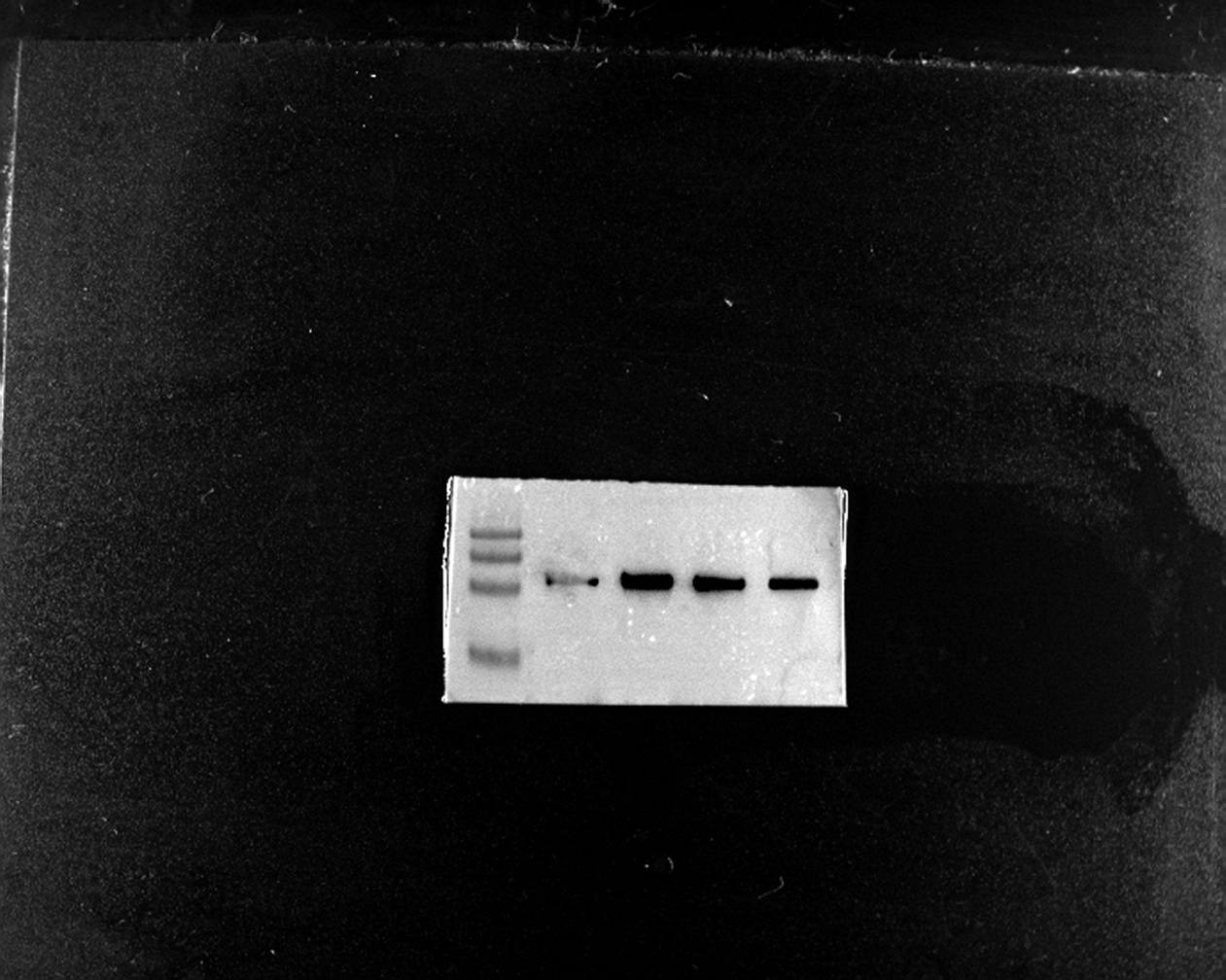


P38


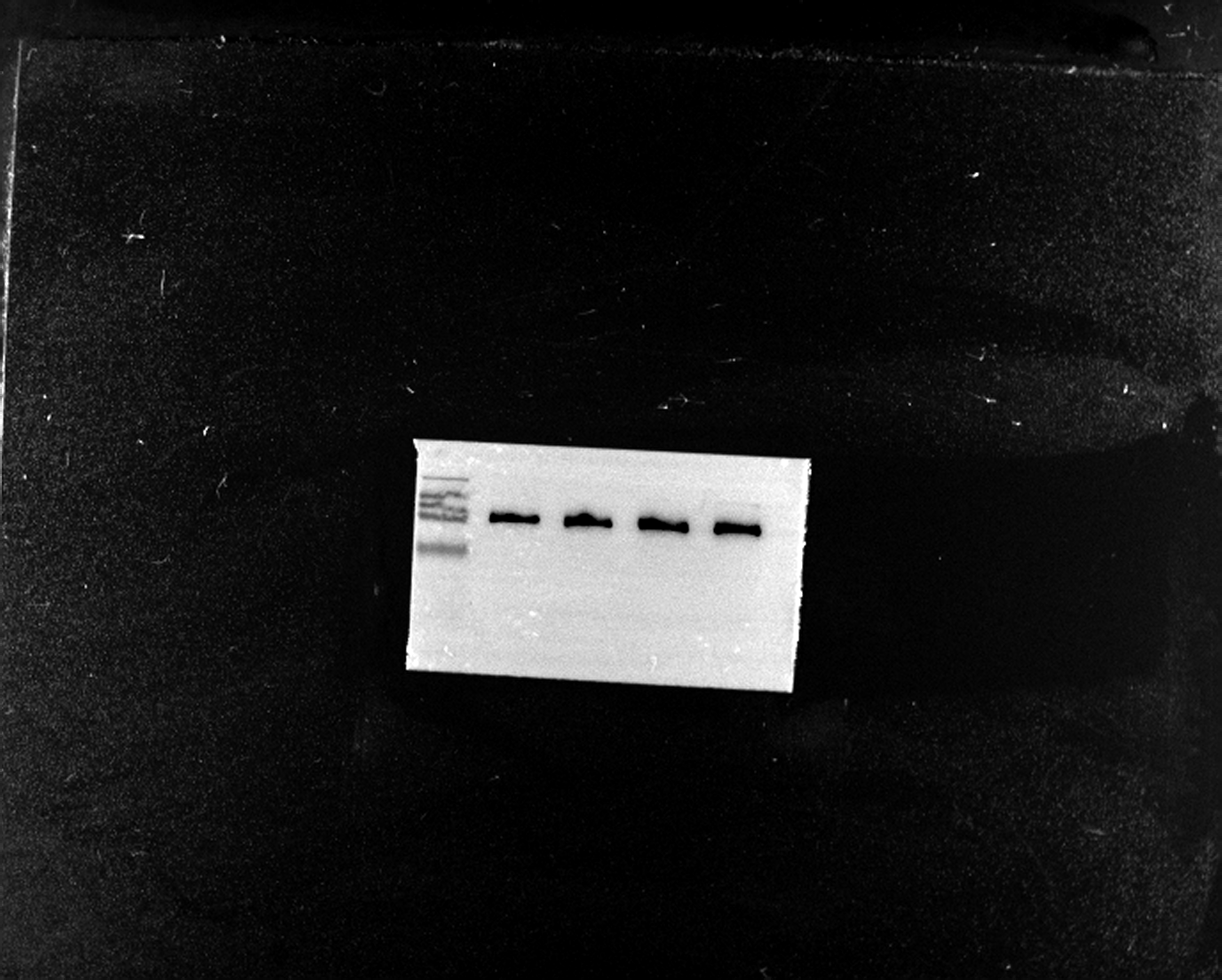


p-JNK


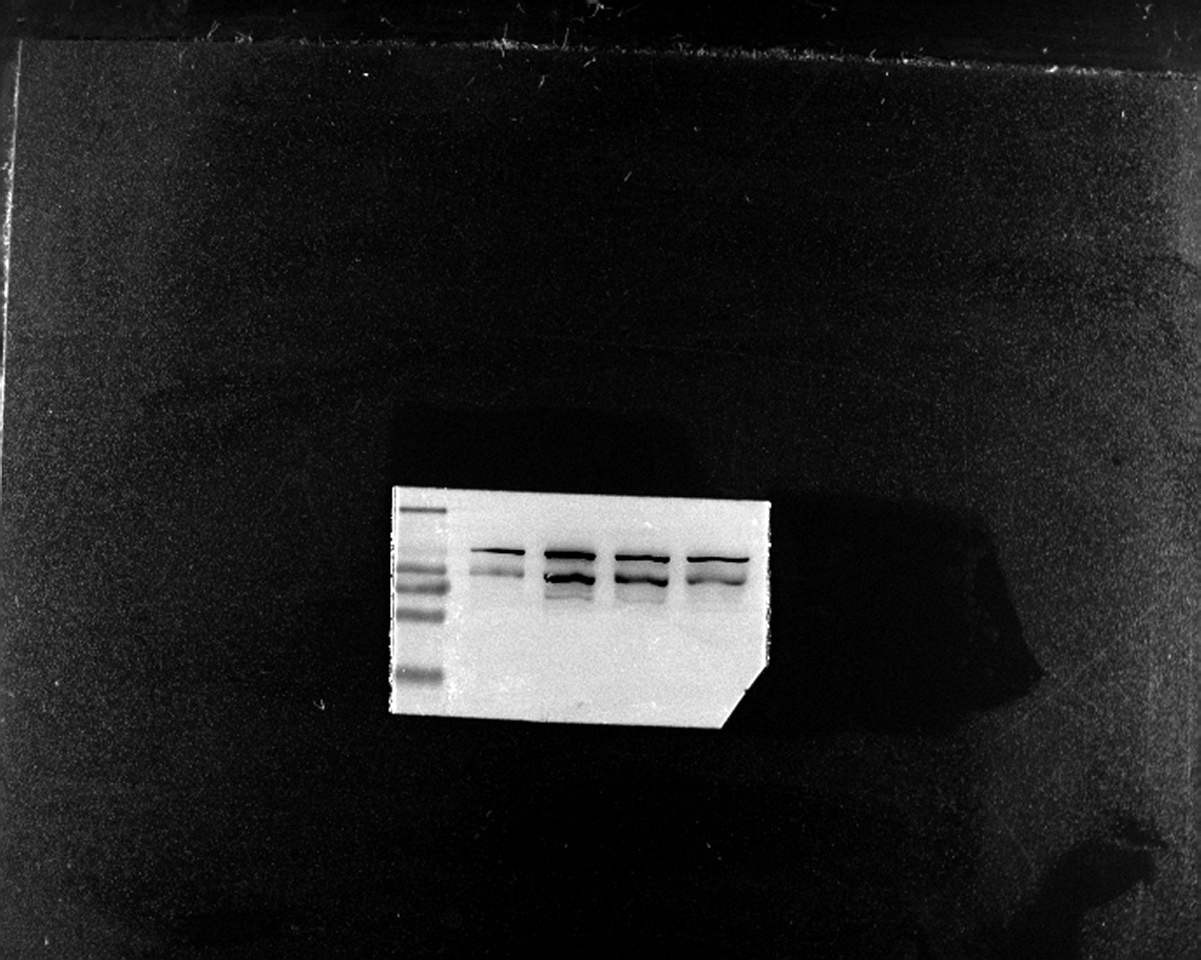


JNK


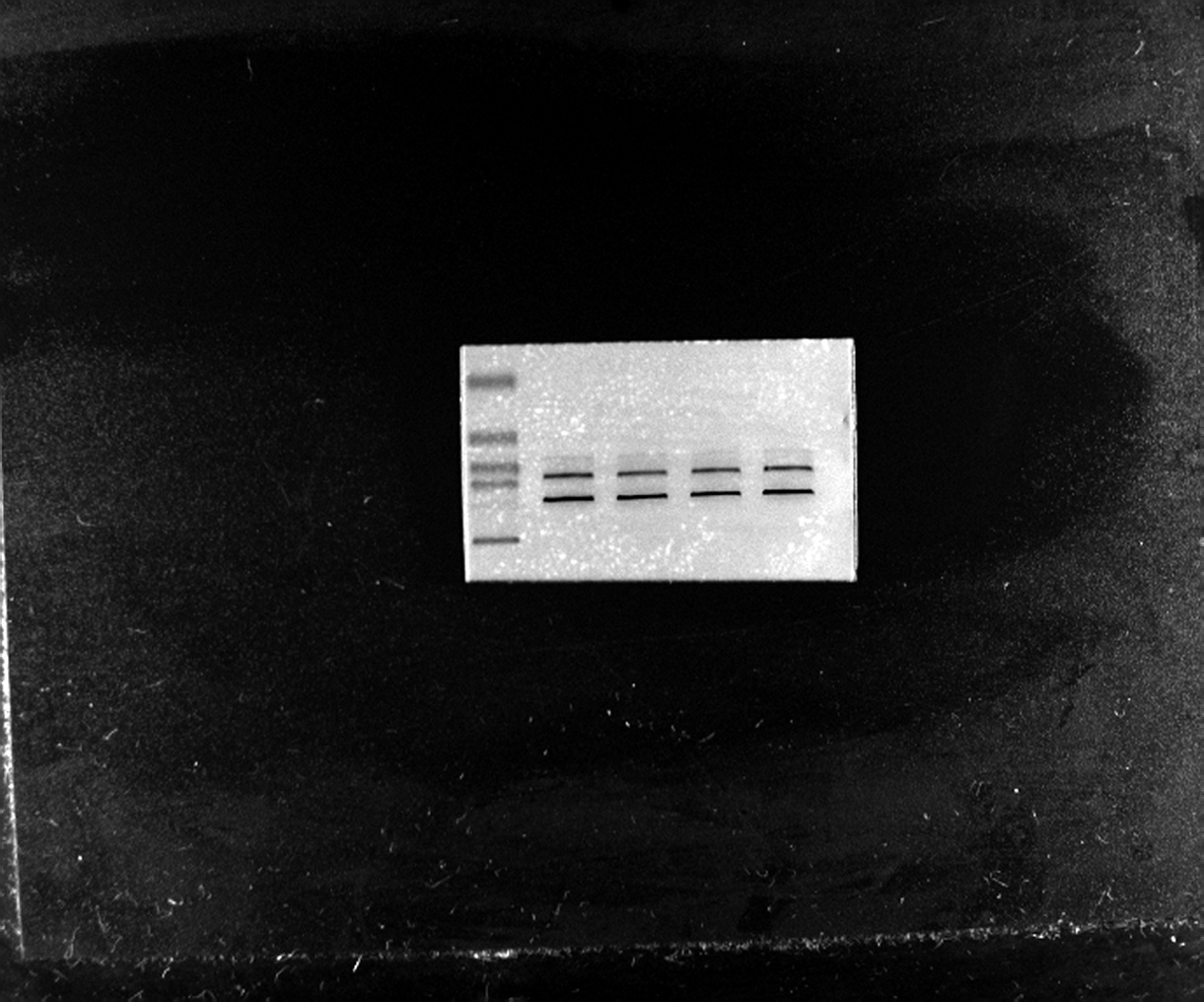


GAPDH


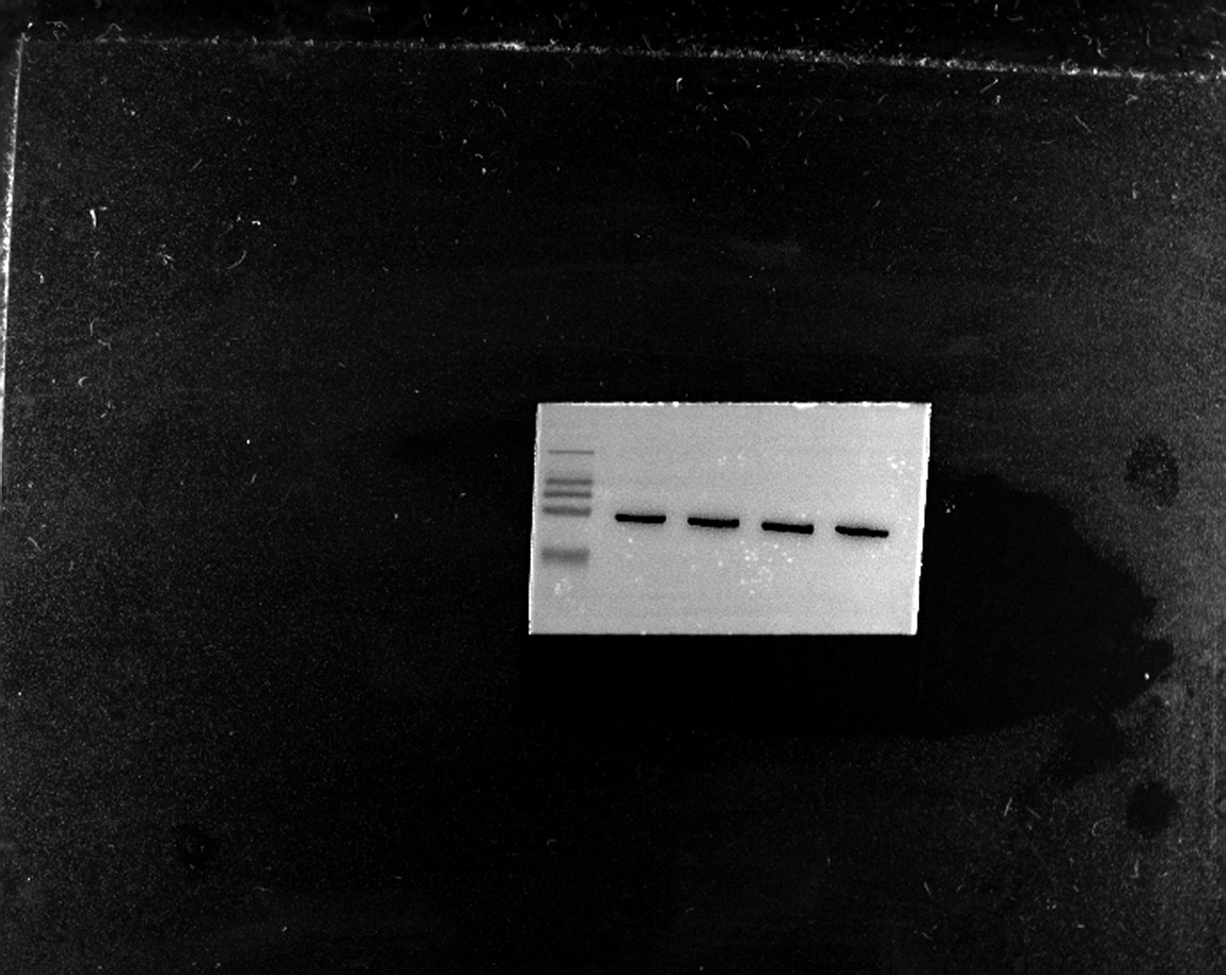


Figure 5A

CTCF


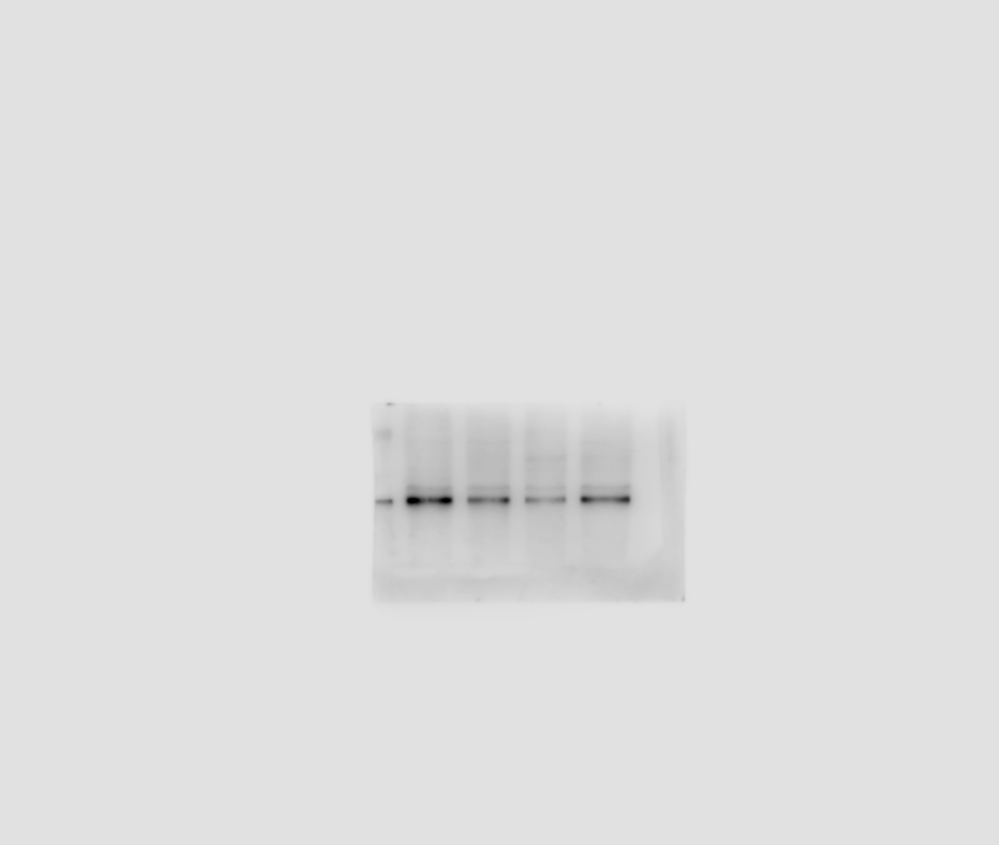


GAPDH


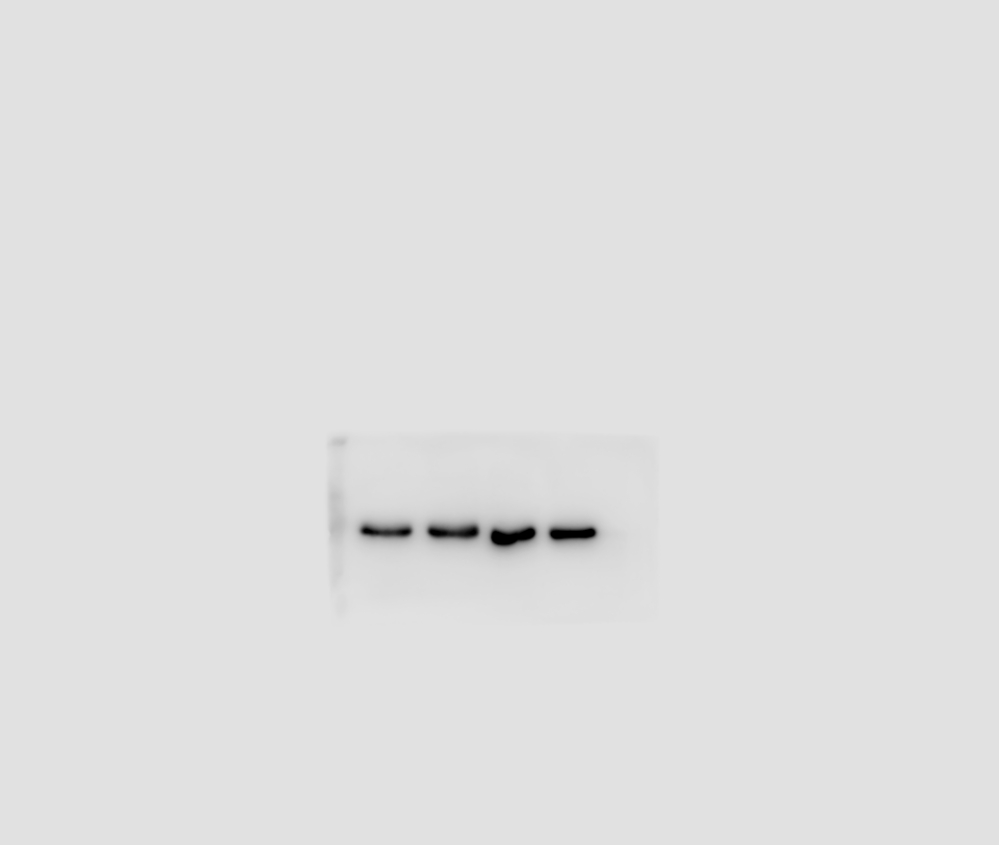


Figure 5B

CTCF


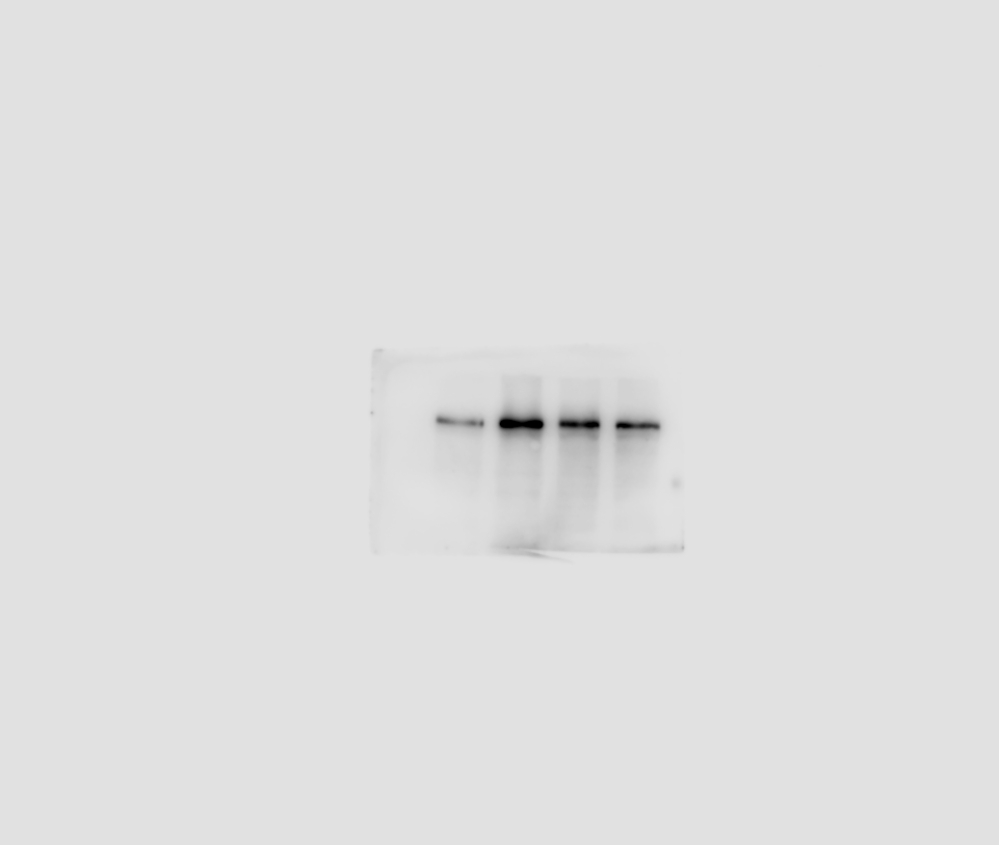


GAPDH


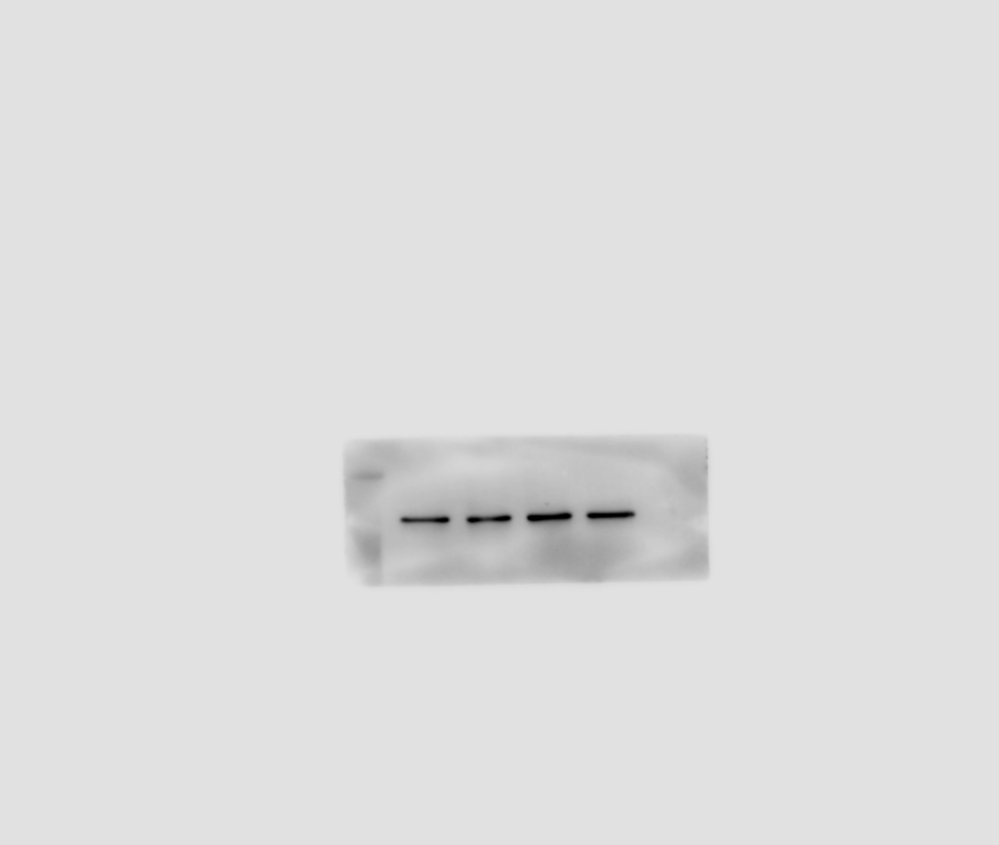


Supplementary Table S1 All differentially expressed genes (DEGs) between NC and si-PDLIM1

Supplementary Table S2 All significantly differentially expressed genes (DEGs) between NC and si-PDLIM1(36 significantly upregulated genes and 104 significantly downregulated genes)
